# Holobiomes in succession: post-glacial microbial communities are structured by hosts, time, and habitat heterogeneity

**DOI:** 10.1093/femsec/fiag107

**Published:** 2026-09-28

**Authors:** Adam Taylor Ruka, Vojtěch Lanta, Samresh Rai, Kateřina Čapková, Thinles Chondol, Inga Hiiesalu, John Davison, Lucie Vančurová, Jan Kučera, Jiří Doležal, Roey Angel, Klára Řeháková

**Affiliations:** Institute of Botany CAS, Třeboň 37901, Czechia; Faculty of Science, University of South Bohemia, České Budějovice 37005, Czechia; Institute of Soil Biology and Biogeochemistry, Biology Centre CAS, České Budějovice 37005, Czechia; Institute of Botany CAS, Třeboň 37901, Czechia; Institute of Botany CAS, Třeboň 37901, Czechia; Department of Environment, Faculty of Environment, University of Jan Evangelista Purkyně, 40096, Czechia; Institute of Botany CAS, Třeboň 37901, Czechia; Institute of Hydrobiology, Biology Centre CAS, České Budějovice 37005, Czechia; Institute of Botany CAS, Třeboň 37901, Czechia; Faculty of Science, University of South Bohemia, České Budějovice 37005, Czechia; Institute of Ecology and Earth Sciences, University of Tartu, 50409, Estonia; Institute of Ecology and Earth Sciences, University of Tartu, 50409, Estonia; Department of Botany, Faculty of Science, Charles University, Prague 128 00, Czechia; Faculty of Science, University of South Bohemia, České Budějovice 37005, Czechia; Institute of Botany CAS, Třeboň 37901, Czechia; Faculty of Science, University of South Bohemia, České Budějovice 37005, Czechia; Faculty of Science, University of South Bohemia, České Budějovice 37005, Czechia; Institute of Soil Biology and Biogeochemistry, Biology Centre CAS, České Budějovice 37005, Czechia; Institute of Botany CAS, Třeboň 37901, Czechia; Institute of Hydrobiology, Biology Centre CAS, České Budějovice 37005, Czechia

**Keywords:** metacommunity, topography, host, primary succession, bacterial–fungal interactions

## Abstract

Glacier forefields provide model ecosystems for studying primary succession, where newly exposed substrates are colonized by microbes associated with lichens, biological soil crusts, mosses, vascular plants, and bulk soil. Collectively, these sources form an interconnected microbial holobiome, yet their roles in structuring bacterial and fungal metacommunities remain poorly understood. We investigated how source identity, moraine age, and topography shape microbial assembly across four glacier forefields in the high-altitude desert of Ladakh, India (northwestern Himalaya). Using a chronosequence approach integrating vegetation surveys, soil physicochemistry, GIS-derived topographic variables, and amplicon sequencing, we found that source identity was the primary driver of microbial diversity and composition. Lichens supported low-diversity communities, whereas mosses, bulk soil, and vascular plants harboured more diverse communities. Bacterial–fungal congruence was significant in all sources except bulk soil, indicating stronger inter-kingdom coupling in host-associated habitats than among soil environments. Source exclusion consistently altered microbial co-occurrence network structure, revealing potential ecological roles among sources, with cryptogams supporting microbial connectivity and vascular plants promoting more specialized interactions. Structural equation modelling further showed that succession indirectly shaped microbial diversity through source colonization and soil development. These findings demonstrate that complementary source-specific ecological strategies organize microbial diversity and interactions during glacier forefield succession.

## Teaser

Distinct habitat types, from mosses to plants and soil, work together to organize microbial diversity and interactions during glacier forefield succession.

## Introduction

An estimated 142 000 km^2^ of habitat around the margins of glaciers in alpine and polar regions will be newly exposed by the end of this century due to accelerated warming (Graversen et al. 2008, Pepin et al. 2015, Zimmer et al. 2022, Nigrelli and Chiarle 2023). These extreme environments, characterized by rocky, nutrient-poor soil and cold, windy conditions, are subsequently colonized by tolerant, pioneer organisms, including microorganisms, cryptogams (mosses and lichens), and vascular plants (Bueno de Mesquita et al. 2017, Darcy et al. 2018, del Moral et al. 2021). This mosaic of macroorganisms and barren soil developing within glacier forefields subsequently provides unique microniches for their associated microbiomes (Bueno de Mesquita et al. 2017, Touchette et al. 2023, Wicaksono et al. 2024). In conjunction with atmospheric and erosional deposition as sources of microbial colonization (Elster et al. 2007, Franzetti et al. 2020, Jensen et al. 2022), these various biotic and abiotic compartments (collectively referred to as ‘sources’ hereafter) constitute a 'holobiome', consisting of positive and negative interactions between microbial metacommunities across various spatial scales (Deveau et al. 2018). Similar to the commonly used term “holobiont”, the holobiome encompasses the microbial interactions and composition throughout an ecosystem as a single ecological unit, recognizing that these communities interact through dispersal, symbioses, and habitat filtering to maintain stable functions and diversity (Garcia et al. 2025). Yet, despite numerous studies assessing the assembly of microbial communities in glacier forefields (Hodkinson et al. 2003, Vimercati et al. 2022, Mandolini et al. 2025), disentangling the relative importance and effects of these sources, such as plants, cryptogams, biological soil crusts (BSCs), and bulk soil, upon the microbial holobiome remains unexplored.

Vascular plants, cryptogams, and BSCs host distinct microbial communities through selective processes such as enhanced nutrient availability and tightly interdependent ecological relationships, while bulk soil acts as a reservoir from which hosts recruit microbial taxa (Cardinale et al. 2006, Ciccazzo et al. 2014, Cania et al. 2020, Chen et al. 2023). Across glacier chronosequences, the distribution of these hosts is shaped by substrate availability and microclimatic conditions. Lichens and BSCs are among the earliest colonizers of newly exposed substrates, contributing to primary production, nutrient cycling, soil stabilization, and stress tolerance through their associated microbiota (Belnap et al. 2003, Cornelissen et al. 2007, Grube et al. 2009, Chamizo et al. 2012, Liu et al. 2024). Mosses also establish early, particularly in moist habitats, and may represent an important but understudied reservoir of microbial diversity (Dickson and Johnson 2014, Bueno de Mesquita et al. 2017, Anderson et al. 2025). As succession proceeds, vascular plants become increasingly dominant and modify microbial communities through root exudation, litter inputs, shading, and the recruitment of mutualistic taxa, coinciding with increases in soil carbon and nutrient availability (Ciccazzo et al. 2014, Kuzyakov and Blagodatskaya 2015, Kawasaki et al. 2021, Ruka et al. 2023). However, climate warming is expected to alter the distribution and recruitment of these colonizing sources across glacier forefields (Wookey et al. 2009). Consequently, understanding the environmental factors that drive source colonization, and how these sources subsequently shape the microbial holobiome, may be critical for predicting the ecological stability of glacier forefield ecosystems.

The chronosequence approach relies on the assumption that sites within a glacier forefield undergo succession under comparable initial environmental conditions, allowing older sites to represent potential future states of younger ones (Wojcik et al. 2021). However, successional trajectories are shaped by the interaction between autogenic processes (e.g. biological interactions, community development, and soil formation) and allogenic drivers (e.g. erosion, extreme weather events, and landscape disturbance) (Matthews 1992). Studies across glacier forefields have revealed relatively predictable successional patterns among plants (Cooper 1916, Negri 1934, Jones and Henry 2003, Dolezal et al. 2008), cryptogams (Favero-Longo et al. 2012), and increasingly microbial communities (Philippot et al. 2011, Zumsteg et al. 2012, Hahn and Quideau 2013, Brown and Jumpponen 2014). However, considerable topographic heterogeneity among moraines may modify these trajectories by creating localized differences in slope, aspect, and concavity that influence microclimate, moisture availability, and resource conditions, ultimately affecting the establishment and persistence of hosts and microbial communities (Solon et al. 2021, Ruka et al. 2023). Therefore, understanding how autogenic factors, such as time since deglaciation and source colonization, interact with allogenic drivers, including topography and climate, is essential for predicting microbial community assembly across glacier forefields.

Furthermore, environmental gradients generated by topography may influence bacterial–fungal interactions by altering microbial community composition within individual sources. Although inter-kingdom relationships between bacteria and fungi remain poorly understood, particularly in alpine and glacier ecosystems, studies from other environments demonstrate that these microorganisms form complex associations through bacterial endosymbiosis, physical attachment to fungal hyphae, and chemical communication mediated by secondary metabolites, volatile organic compounds, and quorum-sensing molecules (Zhao et al. 2017, Steffan et al. 2020). However, these interactions have been studied predominantly in agricultural and temperate ecosystems, leaving their ecological significance in nutrient-poor, rapidly developing glacier forefields largely unexplored (Zhou et al. 2022). Across successional gradients, environmental filtering may therefore shape not only the composition of bacterial and fungal communities but also the extent to which they assemble as coordinated microbial consortia.

In addition to bacterial-fungal congruence, characterizing microbial co-occurrence network structure provides a complementary approach for investigating how environmental gradients and source colonization influence microbial metacommunity assembly. Specifically, the presence or absence of particular sources may restructure microbial networks by altering connectivity among taxa and the formation of highly connected hubs, thereby reflecting differences in the ecological roles of individual sources in organizing microbial metacommunities within the holobiome (Shen et al. 2022, Finck et al. 2026). Network analyses also provide a framework for identifying highly connected taxa that may disproportionately influence community organization, with these taxa often characterized by high connectivity and centrality within microbial networks (Banerjee et al. 2018). While previous studies have focused primarily on within-kingdom network importance, taxa that are both highly connected within microbial networks and associated with bacterial–fungal congruence may represent important taxa for mediating inter-kingdom community organization. Here, we combine eigenvector centrality (Peschel et al. 2021) with associations to bacterial–fungal congruence to identify ‘indicator’ taxa that are structurally important within microbial co-occurrence networks and are also linked to variation in inter-kingdom community coupling. Rather than inferring ecological function, this complementary approach identifies taxa that may play influential roles in microbial community interactions and provides a framework for generating hypotheses about cross-domain interactions during glacier forefield succession.

The heterogeneity of glacier forefields, shaped by interacting abiotic and biotic factors, plays a critical role in the assembly of the microbial holobiome across spatial and successional gradients. Although chronosequence studies have revealed broad patterns of microbial succession (Garrido-Benavent et al. 2020, Vimercati et al. 2022, Ruka et al. 2023, Acuña-Rodríguez et al. 2023), the relative contribution of distinct sources and environmental heterogeneity upon microbial community assembly remains poorly understood. Here, we characterize bacterial and fungal communities associated with distinct sources (lichen, moss, BSC, plant rhizosphere, and bulk soil) across glacier chronosequences in the northwestern Himalaya region of Ladakh, India, an understudied region of microbial biodiversity in the “Third Pole” (Banerjee et al. 2021). Integrating vegetation surveys, molecular sequencing, soil physicochemical analyses, and GIS-derived environmental variables, we investigate how source colonization, successional age, and topographic variation shape the glacier forefield holobiome. Using network analyses and Procrustes tests, we further identify potential indicator taxa and evaluate bacterial–fungal coupling within sources across topographic variation and moraine age. We hypothesized that: (1) bacterial and fungal communities will differ among sources due to source-specific environmental filtering and resource availability; (2) topographic heterogeneity will influence bacterial–fungal community congruence by modifying microclimatic conditions, with effects varying among source types; and (3) microbial metacommunities will be primarily driven by source identity and host-associated processes, with plant colonization exerting strong effects through enhanced soil development and resource availability.

## Methods

### Study sites

The study was performed in the Ladakh region of India, a high-altitude desert located in the northwestern ranges of the Himalaya. Overall, the region often receives less than 100 mm year^−1^ of total annual precipitation due to a strong rain-shadow effect related to orographic barriers. With the region being the intersection of multiple mountain ranges (e.g. Karakoram, Pangong, Greater Himalaya ranges), bedrock material varies considerably in the region from granitic and gneissic, while limestone is common at higher elevations (Phillips 2008, Kowser et al. 2017). Mean annual air temperatures in the regions vary from 1.6 to 10.4 °C while mean annual soil temperature ranges from 0.3 to 7.4 °C (Ruka et al. 2023). The two study locations, Tso Moriri (32°59′N, 78°24′E) and Nubra (34°45′N, 77°35′E) valleys, are separated by 200 kilometers, yet both are near the transition to the Qinghai-Tibetan Plateau. Additionally, they differ in elevation, with glacier tongues terminating in Nubra at 5 150–5400 m.a.s.l. while in Tso Moriri they terminate at 5800 m.a.s.l. Previous studies at the sites investigated environmental stressors and soil phototrophs (Řeháková et al. 2019), plant-facilitation (Dvorský et al. 2013), and plant recruitment (Jandova et al. 2025). Notably, glacier forefield successional pathways of plants, soil chemistry, and soil bacteria communities were studied at the two locations, finding similar bacterial taxa inhabited the environments closest to the glacier tongue (Ruka et al. 2023). These studies have highlighted the importance of elevation, climate, and environmental filtering in shaping plant and microbial distributions, including upper elevational limits of plants (Dolezal et al. 2016), diversity patterns of plant-symbiotic arbuscular mycorrhizal fungi (Hiiesalu et al. 2023), and contrasting microbial successional trajectories across climatic and elevational gradients (Ruka et al. 2023).

### Field work

Transects were performed at four glacier forefields in the Ladakh region during the 2022 field season (15 October 2022 to 29 October 2022). Three glacier tongues of the South Shupka Kunchhang Glacier in Nubra (5400, 5300, and 5150 m) were selected for the study due to geographic proximity, while only one glacier tongue of Chamser Glacier in Tso Moriri (5800 m) was selected due to logistical constraints. Although the sampling design was uneven, previous work demonstrated similar successional patterns in soil bacterial communities across young and old moraines, while plant colonization dynamics in Nubra appeared more stochastic (Ruka et al. 2023). These findings indicate that microbial successional patterns may be relatively consistent across moraines, providing a suitable framework for assessing source-specific and environmental drivers of microbial community assembly across glacier forefields. Starting at the terminus of each glacier tongue, nine plots were established across three moraines with three plots per moraine (*n* = 36). Plots were established along the transect at the front, top, and back side of the moraine to account for topographic variation. Relative age of the moraines was categorized as young, intermediate, and old based on sequential moraines away from the glacier tongue (Fig. 1). Although absolute moraine ages were not determined directly in this study, approximate ages can be inferred from nearby geochronological studies. In the Nubra Valley, Optically Stimulated Luminescence (OSL) dating indicated that moraines approximately 500 m from glacier termini are ∼500 years old, while those around 1000 m away are approximately 1000 years old (Ganju et al. 2018). In contrast, the drier climate of Tso Moriri has resulted in slower glacial retreat and older landforms. Using cosmogenic ^10^Be surface exposure dating, Saha et al. (2019) estimated moraines within 500 m of glacier termini to be 200–500 years old, whereas moraines more than 1000 m away ranged from ~7000 to 23 000 years in age. Accordingly, the young, intermediate, and old moraines in this study likely represent surfaces <500 years, ∼ 500–1000 years, and >1000 years, respectively, with the ages of old moraines differing between locations but representing comparable stages of climax communities in the localized successional trajectories.

**Figure 1:**
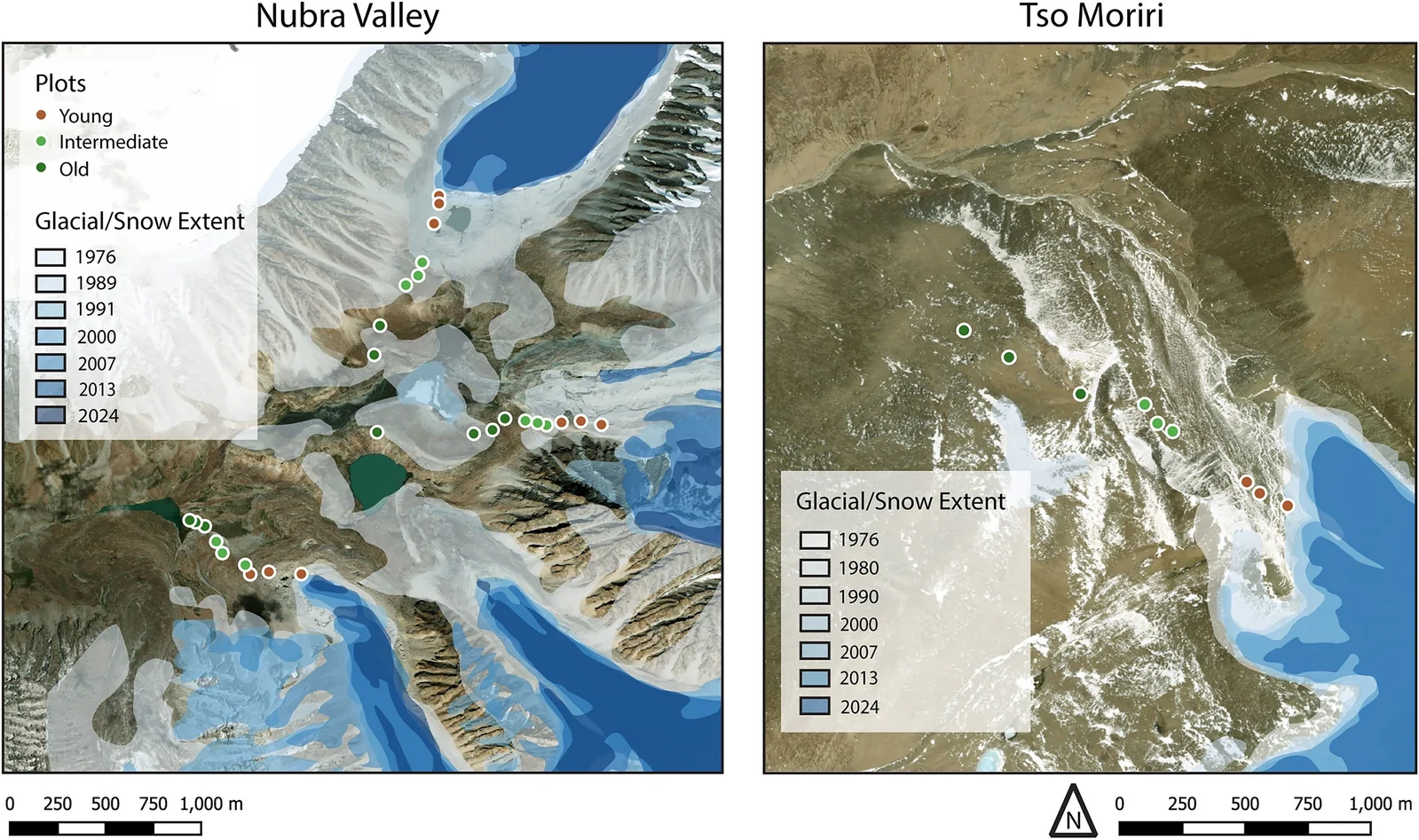
Maps of the studied glacier forefields with past glacial/snow extent based on historical satellite imagery retrieved from the USGS Earth Explorer. Images were selected during the period of minimal snow cover in the Ladakh region (August–October) and optimal visibility. Maps were built in QGIS and projected in Pseudo Mercator using WGS 84 coordinate system.

Within each 1 m^2^ plot, cover (%) was recorded for vascular plants, lichens, mosses, soil crust, stones, and bare soil (Supplementary Table S13). Furthermore, latitude and longitude coordinates, elevation (m.a.s.l.), and distance from the glacier tongue were recorded. Sampling from each plot included a composite bulk soil sample from three subsamples (0–10 cm), lichens, soil crusts, mosses, and fine root samples of the three most dominant plants within the plot. Due to some plants having minimal fine root material, samples were pooled from at least two individuals. From each plot, 3–10 samples in total were collected depending on source occurrence along the transect. Plant species were identified in the field using a local flora guide (Dvorskỳ et al. 2018). All samples were preserved by drying in separate plastic bags with silica gel at ambient temperature (Bainard et al. 2010), while bulk soil samples were air-dried after removing a subsample for molecular analyses. All samples were transported to České Budějovice, Czech Republic for further molecular analyses, identification of mosses and lichens, and soil physicochemistry analyses. Moss and lichen identification methods are detailed in the supplementary material (Appendix 1) (White et al. 1990, Gardes and Bruns 1993, Cubero et al. 1999, Werner et al. 2002, Kučera et al. 2019).

### Soil physiochemistry analyses

Air-dried bulk soil samples from each plot were stored at –20°C until further processing at the Institute of Botany in Třeboň, Czech Republic. Then, soil samples were oven-dried at 80°C and 2 mm sieved before being ground in a mortar. Weight measurements before and after sieving were used to calculate texture (% >2 mm). Soil pH was measured in a 1 : 5 (soil: deionized water) suspension. Soil organic matter content (OM, %) was determined by dry combustion at 450°C for 5 h. Total nitrogen (TN; mg kg^−1^) was quantified using a Lachat QC 8500 automated injection analyzer (three-channel FIA system), with signal detection via the Thermo Scientific Eager Xperience platform (Thermo Fisher Scientific 2016, Sparks 1996). Total phosphorus (TP; mg kg^−1^) was analyzed according to U.S. EPA Method 365.4 and Standard Methods 4500–P G. The major cations (Ca²⁺, Mg²⁺, K⁺, and Na⁺) were quantified by inductively coupled plasma mass spectrometry (ICP-MS) using an ICP-QQQ instrument (Agilent Technologies, Tokyo, Japan) (Kopáček et al. 2004).

### DNA extraction, PCR amplification, and sequencing of sources and soil

DNA from root, lichen, moss, BSC, and bulk soil samples (240 total) was extracted using DNeasy PowerSoil Pro Kit (Qiagen) after adding 100 μl of phosphate buffer for rehydration. On average, 0.2 gm of silica-dried material was used for extraction, and DNA concentration was measured using Quant-iT™ PicoGreen™ dsDNA Assay Kit (Thermo). DNA extracts and three negative controls (blank extractions) were sent to AsperBiogene in Tartu, Estonia, where PCR amplification, library construction, and high-throughput sequencing were performed on an Illumina MiSeq sequencer (Illumina). For fungal barcoding, the ITS2 region was targeted using the fITS7 (5′-GTGARTCATCGAATCTTTG-3′) (Ihrmark et al. 2012) and the ITS4ngsUni (5′-CCTSCSCTTANTDATATGC-3′) (Tedersoo and Lindahl 2016). Although the fITS7–ITS4ngsUni primer pair provides broad coverage of fungal diversity, it exhibits known taxonomic biases, including reduced amplification efficiency for some fungal lineages (e.g. Orbiliales and Mucorales) (Ihrmark et al. 2012). For bacteria, the V4 region of the 16S rRNA gene was amplified using the primers 515F-Y (5′-GTGYCAGCMGCCGCGGTAA-3′) (Parada et al. 2016) and 806R (5′-GGACTACNVGGGTWTCTAAT-3′) (Apprill et al. 2015).

### Bioinformatics

Raw short-read sequencing data (16S rRNA and ITS2) were processed using a pipeline based on DADA2 (Callahan et al. 2016). Primers and linker regions were trimmed using Cutadapt (v3.5) (Martin 2011), followed by quality filtering, merging, denoising, and chimera removal within DADA2 v1.28.0 in R v4.4.1 (R Core team 2025). Non-standard filtering parameters included: filterandTrim function with maxEE = c(2,2) and pseudo pooling. Taxonomic assignment was performed using the SILVA138.2 database (Quast et al. 2013) for 16S and UNITE v10.0 (Abarenkov et al. 2024) for ITS2 sequences. Contaminant ASVs were identified with the *decontam* R package (Davis et al. 2018) using the frequency method (DNA concentration) and the prevalence method (negative controls) and removed prior to downstream analyses. This flagged and removed 165 bacterial ASVs and 93 fungal ASVs. Taxonomic filtering removed Archaea, Mitochondria, Chloroplast, and unclassified sequences from the 16S data and Viridiplantae, Alveolata, Heterolobosa, Ichthyosporia, Metazoa, Rhizaria, Stramenopila, and unclassified sequences from the ITS2 data. Of 238 environmental samples extracted for DNA, 18 were not sequenced (including 13 PCR failures). After DADA2 processing, 220 bacterial and 219 fungal libraries remained. A minimum library size of 1000 reads, singleton removal, and a 1% prevalence threshold (three samples) were then applied. Samples below the library-size threshold were removed (6 bacterial, 5 fungal), and subsequent filtering yielded 211 bacterial samples (mean library size: 20 479; 13 907 taxa; 4 321 109 total reads) and 207 fungal samples (mean library size: 9 739; 2 594 taxa; 2 016 152 total reads). Of these, 201 samples had paired 16S and ITS data. Subsequently, phyloseq objects were created (McMurdie and Holmes 2013) for both bacterial and fungal reads using processed sequence tables, taxonomy, and metadata for bacteria and fungi. To account for variability in the ITS2 region, duplicated taxa, and statistical cohesiveness, both phyloseq objects (for the 16S rRNA and the ITS2) were agglomerated at the genus level. Alpha-diversity metrics (Chao richness and Shannon-Weiner H’ diversity) were calculated using the plot_richness function in the phyloseq package. Last, bacterial and fungal count data were converted to relative abundance values for downstream analyses.

### GIS applications

QGIS 3.42.3 was used for all GIS-derived variables. Topographic variables were extracted using latitude and longitude point features overlaid on the Global Multi-Resolution Topography (GMRT) Synthesis layer, which has a resolution of 30 × 30 m. Topographic Position Index (TPI), slope, and aspect were calculated using the respective tools in QGIS with default settings. TPI indicates the relative position of a location in the landscape, capturing whether it lies in a concave (e.g. valley) or convex (e.g. ridge) terrain. Due to aspect being a circular variable (0° = 360°), it was converted into sine and cosine components to allow for linear statistical analysis. In this transformation, the sine of aspect represents the east–west orientation (positive values = east-facing slopes), while the cosine of aspect represents the north–south orientation (positive values = north-facing slopes).

To assess historical changes in glacial extent and minimum annual snow cover, a geographic database was developed in QGIS by acquiring LANDSAT raster data from the USGS EarthExplorer (U.S. Geological Survey 2024). GeoTIFF layers spanning from the 1970s to the present were downloaded, selecting one image per decade based on cloud-free conditions and minimal snow cover, typically in September or October. Early imagery from 1976 and 1980 had a resolution of 60 × 60 m, while later images had a resolution of 30 × 30 m. Glacial features were digitized from reflectance bands using the polygon drawing tool in QGIS, followed by two iterations of polygon smoothing (Fig. 1). Among these layers, the September 1976 image exhibited the greatest glacial and snow cover extent and was used to generate a categorical variable labeled 'Snow 1976'. All spatial layers were projected using the Pseudo Mercator coordinate system (WGS 84 datum).

### Statistical analyses

All statistical analyses were performed in R v4.4.1. As a preliminary step, redundancy analyses (RDA; R package: vegan) were carried out to evaluate the effect of environmental variables (moraine age, TPI, slope, aspect [sine and cosine], Snow 1976, and elevation) on two sets of response variables: (1) root-mean-squared soil physicochemistry parameters, and (2) isometric log-ratio transformed cover (%) measurements, separately. Each RDA was conducted separately for these two response sets, with models conditioned by location to account for spatial variation and identify broader regional trends. The statistical significance of the full models and individual predictor terms was tested using permutation-based ANOVA with 999 permutations. Furthermore, Spearman rank correlation tests were performed between environmental, plot cover, and soil properties and *P*-values were FDR-corrected.

Bacterial and fungal α-diversity metrics (Shannon-Weiner diversity index, richness) were analyzed using linear mixed-effect models (R package: lmer4) (Bates et al. 2015) with moraine age (young, intermediate, old), source (lichen, soil crust, moss, bulk soil, plant), and location (Tso Moriri and Nubra) as categorical fixed effects. To account for multiple sources being collected from each plot, the plot ID was used as a random effect. Model significance was assessed using analysis of variance via Type II F-tests (R package: car) (Fox and Weisberg 2019). Post hoc comparisons of α-diversity among sources within each moraine age were conducted using aligned rank transformation ANOVA (R package: ARTool) (Wobbrock et al. 2011) followed by pairwise comparisons of estimated marginal means (R package: emmeans) (Lenth 2023), with false discovery rate (FDR) correction applied to p-values. The aligned rank transformation was applied as a non-parametric approach due to unequal sample sizes within moraine ages. Then, bump plots were created to visualize the unique vs. shared genera from each source across moraine ages. Subsequently, the genera shared by all sources across all moraine ages was termed the ‘core’ community and used for network analyses and Procrustes analyses.

For microbial beta-diversity analyses, a center log-ratio (CLR) transformation was applied with a pseudo count of 1e^−6^ added (apart from network analyses where a pseudocount of 1 was added). A PCoA was performed on the CLR-transformed community data using Euclidean distance for both bacteria and fungi (R package: microViz) (Barnett et al. 2021). Beta-dispersion (distance to centroid) was calculated from the resulting ordinations (R package: vegan) (Oksanen et al. 2022), followed by linear mixed-effect model tests as described in the previous paragraph. Permutational multivariate analysis of variance (PERMANOVA) analyses (999 permutations) were paired with PCoA analyses using moraine age, source, and location as explanatory variables (R package: vegan). Because plots are nested within location and moraine age, two complementary models were fitted for the combined (all-source) analyses: (i) the same predictors with permutations restricted to within plots (strata = Plot ID), which provides a more conservative test of location and moraine age by preventing free exchange of samples among plots; and (ii) an unrestricted PERMANOVA that additionally included Plot ID as a fixed effect, allowing comparison of term significance with and without plot-level blocking. Upon finding that source identity explained the most variation in bacterial and fungal community composition, subsequent PERMANOVA analyses were performed on separate sources with moraine age and location as explanatory variables. Plot ID was included as a fixed effect for sources which had multiple samples per plot (lichens, mosses, and plants). *P*-values were adjusted using FDR correction for multiple comparisons. Last, composition bar plots were generated based on relative abundance of pooled samples of all shared taxa and individual sources.

Patterns of coupling or decoupling between bacterial and fungal communities were assessed using Procrustes analysis (R package: vegan) (Peres-Neto and Jackson 2001). For each analysis, bacterial and fungal genus-level CLR matrices were ordinated separately using redundancy analysis conditioned by location [clr ∼ 1 + Condition(Location)], and the constrained scores were compared with symmetric Procrustes rotation [protest(), 999 permutations]. Analyses were performed on all samples combined and separately for each source; sample sizes are reported in Supplementary Material (Appendix 2). Sample-level Procrustes residuals (higher values indicate greater bacterial–fungal decoupling) were rank-transformed, z-scored and related to environmental variables using linear models. The effect of Plot ID was also tested using ANOVA to determine the potential effect of multiple samples coming from the same plot. An ordination based on mean bacterial and fungal Procrustes site scores (consensus coordinates) was visualized with the top two genera for bacteria and fungi with negative and positive associations with Procrustes residuals (8 total taxa). A repeated sensitivity analysis (unconditioned by location) is included in Supplementary Methods (Appendix 1).

Genus-level bacterial and fungal co-occurrence networks were inferred with NetCoMi (R package: NetCoMi) (Peschel et al. 2021) on the core community (defined above), separately for each Glacier x Moraine unit (glacier identity × moraine age class; 3 plots per unit). Units with fewer than eight samples were excluded; units with more than eight samples were analyzed using bootstrap subsampling (ten iterations, parameters averaged). The workflow was repeated under leave-one-source-out exclusion treatments (all sources combined vs. exclusion of lichen, moss, soil crust, bulk soil, or plant). Global network parameters from these treatments were compared using linear mixed-effect models with source-exclusion treatment and moraine age as fixed effects and Glacier x Moraine identity as a random effect (R packages: lmer4, car). Model significance was assessed using analysis of variance via Type II F-tests (R package: car) with FDR-adjusted post-hoc pairwise comparisons of estimated marginal means (R package: emmeans). Network construction parameters, bootstrap averaging, calculations of high-centrality taxa, and a sensitivity analysis using 20 samples per network are detailed in Supplementary Material (Appendix 1).

To evaluate taxa that were structurally important in co-occurrence networks and associated with bacterial–fungal congruence, we used a two-step screening approach. First, high-centrality genera were identified as those with eigenvector centrality ≥0.95 in more than three networks (including exclusion treatments) and present as network nodes in both locations. Second, Procrustes-residual indicator genera were identified as those with significant Spearman correlations (*P* < .05) between relative abundance and sample-level Procrustes residuals, with a maximum relative abundance >1% within the relevant source. Overlap between these two sets was summarized separately for bacteria and fungi by source. High-centrality genera were ranked by the number of networks in which they met the eigenvector-centrality threshold, with additional stratification by moraine age class and location where applicable. For genera meeting both criteria, follow-up Spearman rank correlations between relative abundance and each environmental variable were performed and FDR-adjusted.

Directional relationships among environmental gradients, source colonization, soil development, and microbial diversity were examined using path analysis (structural equation modelling, SEM; R package: lavaan v0.6) (Rosseel et al. 2012). For each group of variables, PCAs conditioned by location were performed and first axis scores were extracted for each plot. The hypothesized succession pathway was: environmental predictors (Distance from glacier terminus; TopoPC1) → source colonization (VegPC1) → soil development (SoilPC1) → microbial diversity (bacterial and fungal metacommunity richness; bacterial and fungal metacommunity Shannon H’). Metacommunity richness and H’ index were estimated separately for bacteria and fungi genera by pooling read counts across all samples within each plot, and rarefying each pooled plot to the minimum pooled library size (bacteria: 36 876; fungi: 2 729). To reduce stochastic variation from a single subsample, rarefaction was repeated 999 times; observed genus richness and Shannon H′ were calculated on each rarefied matrix and averaged across iterations to give one value per plot. Direct effects were tested in the full model, followed by a trimmed model which retained paths with a *P*-value < .1. All continuous predictors were z-scored prior to modelling. Path coefficients were standardized, and standard errors and 95% confidence intervals were estimated using parametric bootstrap (1000 resamples) with bias-corrected and accelerated (BCa) intervals. Global model fit was evaluated using the chi-squared statistic, Comparative Fit Index (CFI ≥0.95), Root Mean Square Error of Approximation (RMSEA ≤0.08), and Standardised Root Mean Square Residual (SRMR ≤0.08). To assess the effect of location upon the pathways, a sensitivity analysis was performed.

## Results

### Environmental variables, cover measurements, and soil physicochemistry variables

Preliminary RDAs showed a predominant effect of moraine age (*P* < .01) upon both soil physicochemistry parameters and cover measurements from the studied transects (Supplementary Figs S1, S2). The amount of constrained variation was higher for cover measurements (50.2%) than for soil parameters (34.6%). However, the amount of conditioned variation by location was higher for soil parameters (23.8%) than for cover measurements (5.4%). On old moraines, graminoid and forb cover increased with higher OM, TN, and other micronutrients such as Ca, K, and Mg. Young moraines had higher coverage of stones and coarse-grained soil texture. Among the remaining environmental variables, TPI was often related to the second axis and negatively correlated with soil crust cover, bare soil cover, while being positively correlated with increasing pH. Taken together, these results suggest a stronger difference in soil properties between the two study locations, while cover measurements differed less, although both RDAs demonstrate that moraine age is the primary factor determining their variation.

Individual Spearman rank correlations revealed similar relationships between environmental variables and source colonization patterns (Supplementary Figs S3–S5). Among cover measurements, lichen cover was negatively associated with the 1976 snow extent proxy, while moss cover showed a negative relationship with aspect (sine), suggesting sensitivity to local topographic conditions. Moss and graminoid cover decreased with elevation, whereas lichen, forb, and graminoid cover increased with both distance from glacier and moraine age (Supplementary Fig. S3). Soil physicochemical properties showed fewer significant relationships, with variation primarily associated with distance from glacier, moraine age, and elevation (Supplementary Fig. S4). In particular, pH, Mg, K, and Ca decreased with elevation, reflecting stronger location-specific differences in soil conditions, while OM, Total N, K, and Ca increased with moraine age. Relationships between source cover and soil properties were considerably stronger, with increasing concentrations of Mg, K, and Ca positively associated with lichen, moss, forb, and graminoid cover (*P_adj_* < .05; Supplementary Fig. S5). Similarly, OM and Total N increased with the cover of lichens (*P*_adj_ < .05), mosses (*P*_adj_ < .05), forbs (*P*_adj_ < .001), and graminoids (*P*_adj_ < .001), highlighting the progressive coupling between vegetation establishment and soil development along the glacier forefield chronosequence.

### Sources of microbial diversity

Among the five sources, plants were most frequently sampled source along the studied chronosequences (*n* = 94 samples) (Supplementary Table S1). In total, rhizosphere microbial communities were assessed from 31 plant species, including *Carex borii* (11), *Waldhemia tridactylites* (9), *Thylacospermum caespitosum* (8), *Saussarea glacialis* (7), and *Carex sagaensis* and *Potentilla palmirica* (both 6), as the most frequently sampled species. Lichens were the second most common sampled source (7 fungal hosts identified; 36 total) with *Xanthoria elegans* (18) and *Gyalolechia bracteata* (4) being particularly prevalent. BSCs followed with 35 successfully sequenced samples. Mosses were second to last with 28 microbial communities sequenced and 10 moss species identified (*Distichium capillaceum and Bryum* spp. being the most prevalent). Bulk soil samples (21) were the least successful in sequencing, particularly in early moraine plots, highlighting the low microbial biomass in these soils. Downstream single-marker analyses used slightly fewer samples after filtering steps (e.g. plant 16S rRNA samples, *n* = 92).

### Bacterial and fungal α-diversity

Genus-level bacterial and fungal α-diversity metrics were primarily influenced by source; however, moraine age also had a significant effect on both kingdoms (Supplementary Table S2). For bacterial communities, source strongly affected Chao richness (F = 47.34, *P* < .001) and Shannon diversity (F = 75.51, *P* < .001), and moraine age also had a significant effect on both metrics (Chao: F = 6.35, *P* = .005; Shannon: F = 4.77, *P* = .016) (Supplementary Table S2). Amongst the sources, bacterial Chao richness and Shannon diversity (H’) were consistently lowest in lichen samples across all moraine ages. In contrast, soil crusts, mosses, bulk soil, and plants exhibited similar diversity in young moraines, while mosses and plants showed higher richness and diversity in intermediate and old moraines (Fig. 2A). For fungal communities, source strongly affected Chao richness (F = 66.29, *P* < .001) and Shannon diversity (F = 34.48, *P* < .001), and moraine age also had a significant effect on both metrics (Chao: F = 10.09, *P* < .001; Shannon: F = 7.12, *P* = .003) (Supplementary Table S2). Lichens and soil crusts had the lowest fungal Chao richness and H’ diversity across all ages, while plants, mosses, and bulk soil samples showed progressively higher values (Fig. 2B). Bump plots illustrating unique and shared genera among sources revealed that shared genera were predominant throughout the glacial chronosequence. The proportion of shared bacterial genera increased from 78% to 84%, and shared fungal genera from 47% to 57%, across moraine ages (Fig. 2D). Among sources, plants hosted the highest proportion of unique bacterial genera, while bulk soil, followed by plants, harbored the most unique fungal genera across all moraine ages. Although these patterns suggest substantial exclusivity of microbial genera by source identity, a large fraction of sequencing reads belonged to the ‘core’ microbiome, defined as genera shared across all moraine ages. Specifically, the core comprised 93.4% (4 037 426) of bacterial reads and 83.2% (1 677 114) of fungal reads. These findings present both the niche-specific associations of certain taxa and the widespread distribution of others, resulting in distinct patterns of microbial α-diversity across glacial successional stages in different sources.

**Figure 2:**
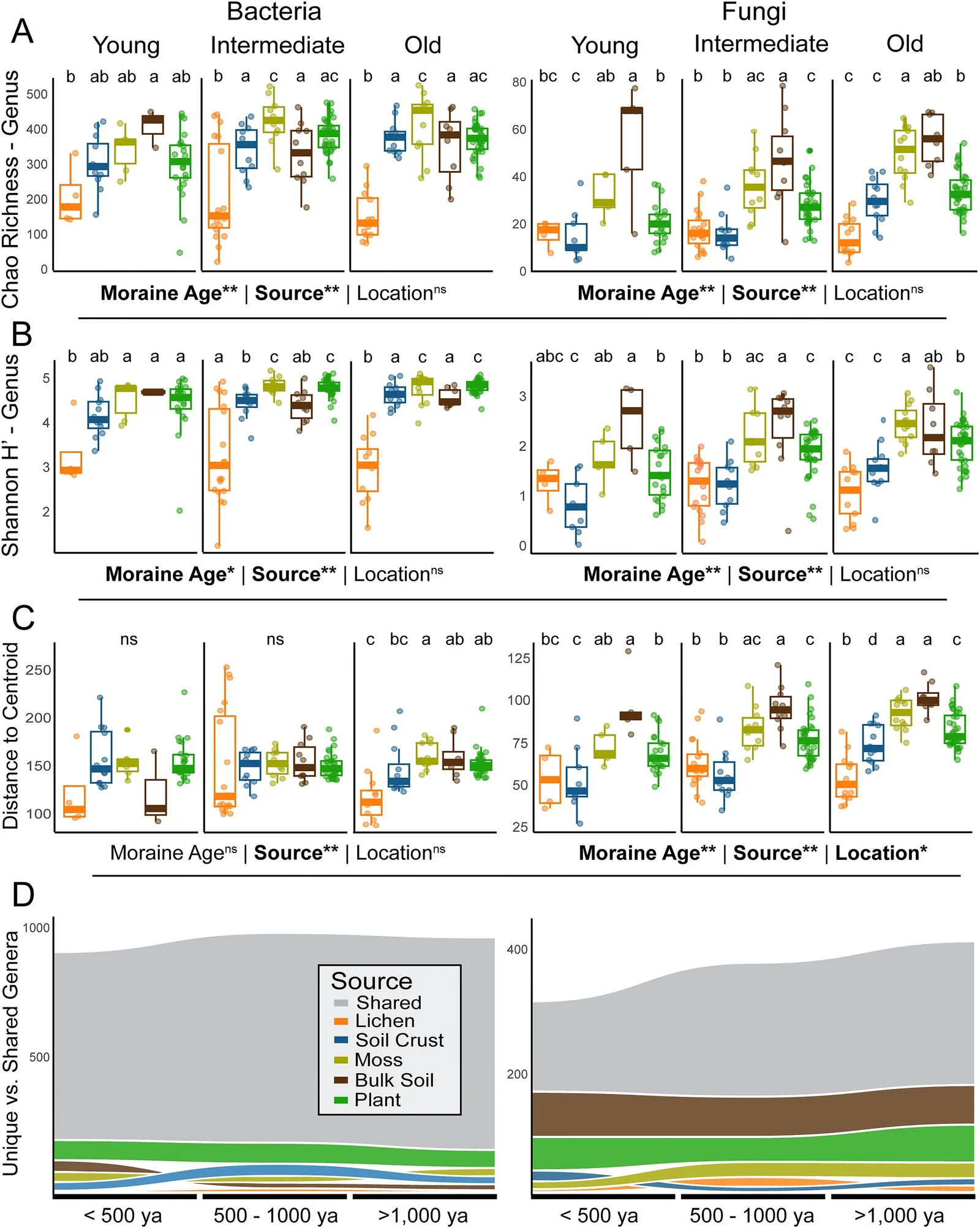
Boxplots (median and quartiles) of bacterial and fungal Chao richness (A), Shannon-Weiner (H’) diversity index (B), and beta-dispersion (C) at the genus level for each respective source across three categorized moraine ages (young, intermediate, old). Linear mixed-effect models were performed to test the effect of moraine age, source, and location with individual plots as a random factor (* *P*_adj_ < .05, ** < .01, *** < .001). Bump plots (D) show the number of unique vs. shared genera found in each source across the categorized moraine ages (Young, Intermediate, Old). Letters indicate post hoc tests between sources within moraine ages.

### Bacterial and fungal β-diversity

Community composition of both bacterial and fungal taxa followed similar trends, with source, moraine age, and location all significantly influencing variation, as shown by PERMANOVA analyses (Fig. 3A). Source had the strongest effect, explaining 20.9% of variation in bacterial communities and 10.0% in fungal communities. Among bacteria, moraine age accounted for more variation (*R*^2^ = 2.1%) than location (1.4%), whereas in fungal communities, location explained slightly more variation (3.2%) than moraine age (2.8%). A complementary PERMANOVA including Plot ID as a fixed effect (no strata) confirmed a significant among-plot contribution to community composition in both bacteria (*F* = 1.49, *R*² = 16.5%, *P* = .001) and fungi (*F* = 1.73, *R*² = 21.0%, *P* = .001). Source remained the strongest predictor, and moraine age and location remained significant (Supplementary Table S3). When analyzed within each source, bacterial community composition was significantly influenced by moraine age in moss and plant samples, and by location in soil crust, bulk soil, and plant samples. For fungal communities, moraine age significantly affected soil crust, moss, and plant samples, while location influenced communities in lichens, mosses, bulk soil, and plants (Supplementary Table S3). Beta-dispersion linear mixed-effect models showed source identity had the most significant effect upon bacterial communities. In contrast, fungal beta-dispersion was more dynamic, being affected by source identity and location, while generally increasing in older moraines (Fig. 2C, Supplementary Table S4).

**Figure 3:**
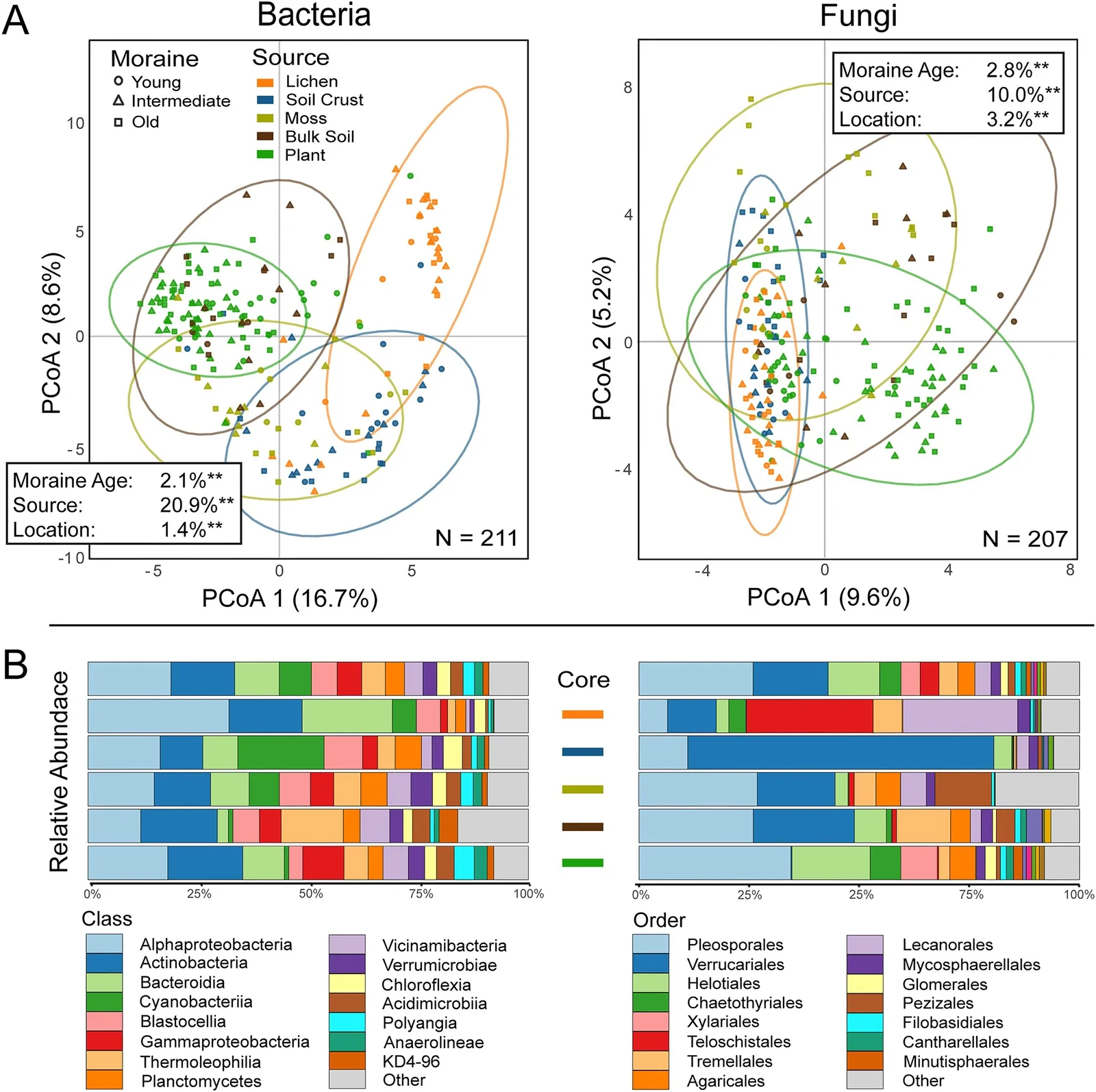
Principal Coordinates Analysis (PCoA) of bacterial (*n* = 211) and fungal (*n* = 207) genus-level composition, grouped by source and moraine age (A). Data was centered log-ratio (CLR) transformed and analyzed using Euclidean distance. Ellipses represent 95% confidence intervals for each source. PERMANOVA analyses were performed using the same transformation and distance calculations with individual plots as a random factor. Compositional bar plots (B) show the pooled relative abundance of higher taxonomic classifications for each respective source and shared genera across all sources (* *P*_adj_ < .05, ** < .01).

### Bacterial and fungal community congruence

Procrustes analyses used only samples retained in both markers (plant *n* = 89; lichen 34; soil crust 31; moss 26; bulk soil 21). Among the Procrustes analyses of all samples and individual sources, significant bacterial–fungal congruence was detected for all datasets except bulk soil (permutation test, *P* < .05; Supplementary Table S5). Procrustes correlation was highest when all samples were analysed together (*R* = 0.73), followed by plant (0.57), moss (0.46), lichen (0.44), and soil crust (0.44) samples. Rank-based linear models relating Procrustes residuals to environmental variables were largely non-significant, with the exception of a positive relationship between elevation and residuals across all samples (regression coefficient = 0.15, *P* = .03), indicating lower bacterial–fungal congruence at higher elevations. The Procrustes ordination (Fig. 4) further showed that taxa associated with higher residuals (lower congruence) were primarily characteristic of lichen, moss, soil crust, and bulk soil samples (bacterial taxa: *Acidiphilum* and an unclassified Acetobacteraceae genus; fungal taxon: *Endocarpon*), whereas taxa associated with lower residuals (higher congruence) were predominantly plant-associated (bacterial taxa: *Kineosporia* and an unclassified Birii41 genus; fungal taxa: *Dominikia* and an unclassified Glomeraceae genus). Although the overall analysis was likely influenced by the greater number of plant samples, the source-specific Procrustes analyses corroborated this pattern, demonstrating that plant-associated communities exhibited the highest bacterial–fungal congruence while other sources also remained significant.

**Figure 4:**
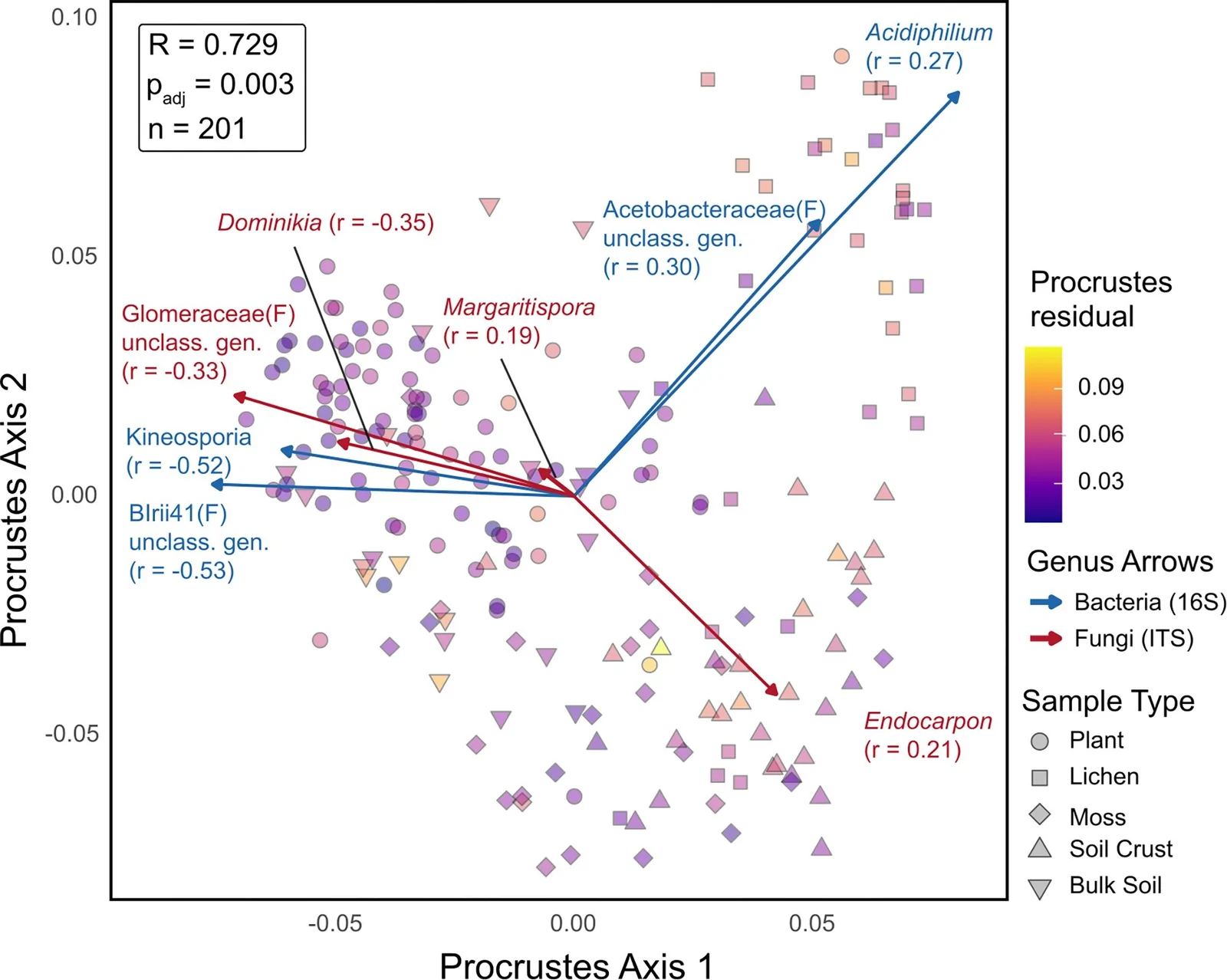
Procrustes ordination based on the mean consensus scores (mean of bacterial and fungal Procrustes scores) for all samples. The two bacterial and two fungal taxa with the strongest positive and negative Spearman correlations (r) between relative abundance and Procrustes residuals are overlaid to illustrate taxa associated with lower and higher bacterial–fungal congruence across sources.

### Microbial co-occurrence patterns and source exclusions

Network analyses showed that global co-occurrence network parameters were sensitive to source exclusion, with contrasting responses between bacterial and fungal communities. A total of 65 bacterial and 65 fungal networks were constructed across all source exclusion treatments and moraine ages. For bacterial networks, only source exclusion treatment remained significant after FDR correction, affecting all eight global network parameters (LMER: *P*_adj_ < .01; Supplementary Table S7). The three parameters with the largest χ² values were visualized (Fig. 5) with post hoc comparisons showing source-specific responses. Average path length increased when mosses and bulk soil were excluded but decreased following plant and soil crust exclusion. Natural connectance decreased following lichen and soil crust exclusion, while positive edge percentage decreased after lichen exclusion but increased following soil crust exclusion.

**Figure 5:**
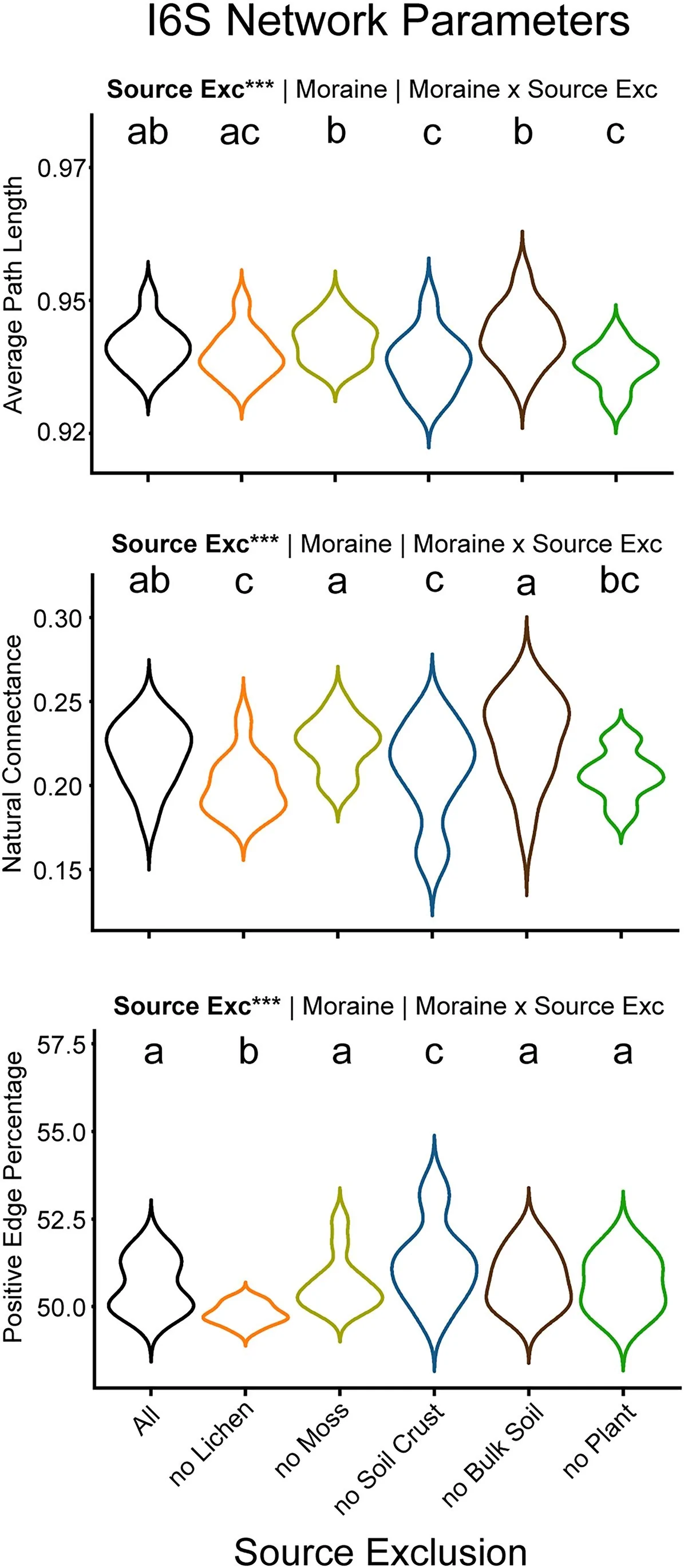
Violin plots showing bacterial global network parameters following separate source exclusion treatments. Network parameters were analyzed using linear mixed-effects models with Glacier x Moraine unit included as a random effect, and *P*-values were adjusted using the false discovery rate (FDR) correction (*** *P*_adj_ < .001). Pairwise comparisons among source exclusion treatments were performed using estimated marginal means with post hoc contrasts.

In fungal networks, source exclusion significantly affected seven of eight network parameters, whereas moraine age significantly influenced average path length and edge connectance (Supplementary Table S7). Plant exclusion produced the strongest changes, increasing average node dissimilarity while decreasing natural connectance and positive edge percentage (Fig. 6). Old moraines had lower average path length and higher edge connectance than younger moraines. Overall, source exclusion altered bacterial and fungal network parameters, whereas moraine age influenced only fungal networks, suggesting greater sensitivity of fungal co-occurrence structure to successional stage.

**Figure 6:**
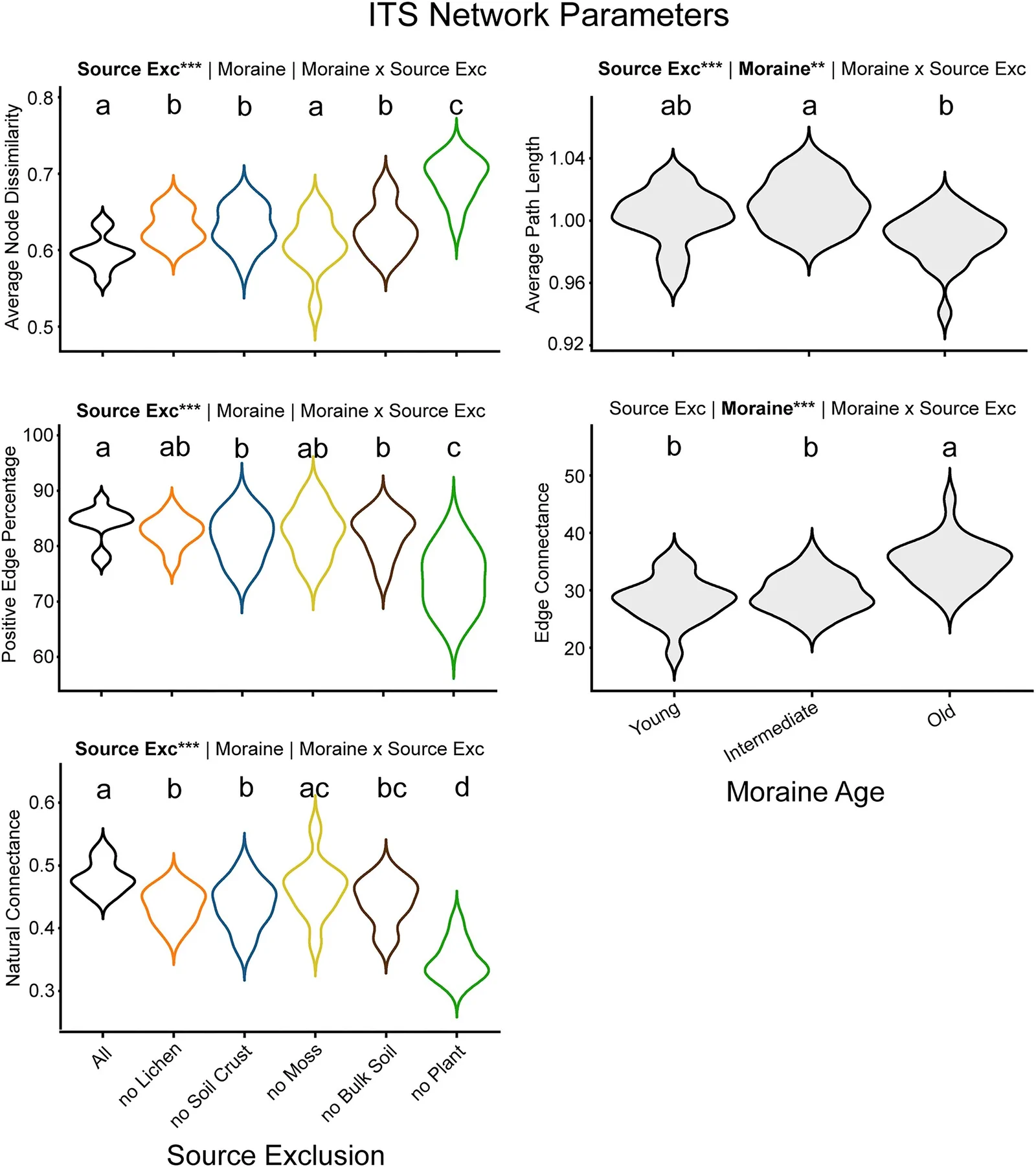
Violin plots showing fungal global network parameters following separate source exclusion treatments and moraine ages. Network parameters were analyzed using linear mixed-effects models with Glacier x Moraine unit included as a random effect, and *p*-values were adjusted using the false discovery rate (FDR) correction (** *P*_adj_ < .01, *** < .001). Pairwise comparisons among source exclusion treatments were performed using estimated marginal means with post hoc contrasts.

### Indicator bacterial and fungal taxa

Following our dual-criteria approach, six bacterial and eight fungal taxa were identified as indicator taxa, representing community members strongly associated with within- and inter-kingdom community composition. All bacterial indicator taxa were negatively correlated with Procrustes residuals, indicating greater relative abundance where bacterial and fungal community composition was more congruent (Table 1). Four bacterial taxa were primarily associated with plant rhizosphere samples and two with bulk soil. Additionally, four bacterial taxa were high-centrality nodes (mean eigenvalue >0.95) in both locations. These high-centrality taxa occurred across different successional stages, with *Vicinamibacter* and an unclassified 67–14 genus present across all moraine ages, three taxa associated with intermediate–old moraines, and an unclassified Microscillaceae genus associated with young moraines. Four taxa presented negative associations (Spearman rank correlation tests) between relative abundance and aspect (cosine; north-facing slopes), while two also declined with increasing elevation, indicating greater relative abundance at the lower-elevation Nubra sites.

**Table 1:** Indicator bacterial taxa identified across glacier forefields in the Ladakh region of northwest India using a dual-criteria approach integrating within-kingdom network centrality and inter-kingdom associations (Procrustes analyses).

Taxon	Dominant source	Max RA (%)	Network Parameters			Procrustes Analysis		Environmental Associations (FDR-corrected)
			% High-centrality (Total)	Moraine Age	Location	Source	Residuals x RA (Spearman rho)	
*Vicinamibacter*	Plant	3.49	12.3 (8)	All	Both	All	−0.471***	Elevation (−)***; Aspect cos (−)*
Unclassified Subgroup 17 genus	Plant	2.50	10.8 (7)	Intermediate–Old	Both	All	−0.267***	Aspect cos (−)***
Unclassified Pedosphaeraceae genus	Plant	1.23	7.7 (5)	Intermediate–Old	Nubra	All	−0.394***	Elevation (−)*; Aspect cos (−)**
Unclassified Microscillaceae genus	Plant	3.93	6.2 (4)	Young	Both	All	−0.461***	None
Unclassified 67–14 genus	Bulk soil	10.39	6.2 (4)	All	Both	All	−0.195*	None
Unclassified OM190 genus	Bulk soil	1.00	6.2 (4)	Intermediate–Old	Nubra	All	−0.253**	Aspect cos (−)**

Taxa are ranked by the number of networks in which they were identified as high-centrality nodes (65 total networks). Environmental associations represent significant FDR-corrected relationships between genus relative abundance and environmental variables; the direction of relationships is indicated by the sign of Spearman’s rho (* *P*_adj_ < .05, ** < .01, *** < .001).

Fungal indicator taxa exhibited stronger environmental associations than bacterial taxa. As with bacteria, all fungal indicator taxa were negatively correlated with Procrustes residuals and were primarily associated with plant rhizosphere or bulk soil samples (Table 2). Six of the eight taxa were high-centrality nodes only in Nubra, whereas *Keissleriella* and an unclassified Glomeraceae genus were high-centrality taxa in both regions. Elevation and distance from the glacier were significantly associated with the relative abundance of five taxa, while north-facing aspect was associated with four taxa. Most high-centrality taxa occurred on young and old moraines, with only *Paraphoma* showing a preference for intermediate moraines. Together, these results suggest that bacterial indicator taxa were primarily structured by location and aspect, whereas fungal indicator taxa exhibited stronger associations with more topographic features and successional stage. Furthermore, the stability of high-centrality bacterial and fungal indicator taxa across networks constructed with 8 versus 20 samples is reported in Supplementary Tables S8 and S9.

**Table 2:** Indicator fungal taxa identified across glacier forefields in the Ladakh region of northwest India using a dual-criteria approach integrating within-kingdom network centrality and inter-kingdom associations (Procrustes analyses).

Taxon	Dominant Source	Max RA (%)	Network Parameters			Procrustes Analysis		Environmental Associations (FDR-corrected)
			% High-centrality (Total)	Moraine Age	Location	Source	Residuals x RA (Spearman rho)	
*Keissleriella*	Plant	46.6	12.3 (8)	Young + Old	Both	All	-0.196**	Elevation (−)***, TPI (+)***, Slope (+)***
Unclassified Glomeraceae Genus	Plant	9.74	10.8 (7)	Young + Old	Both	All	-0.334***	Elevation (−)***, Aspect (cos) (−)**, Distance from glacier (+)**
*Paraphoma*	Bulk soil	94.8	7.7 (5)	Intermediate	Nubra	All	-0.183**	Slope (+)*, Aspect (cos) (−)***, Distance from glacier (+)**
Unclassified Diversisporaceae genus	Bulk soil	13.3	7.7 (5)	Young + Old	Nubra	Bulk soil	-0.508*	None
*Cyphellophora*	Plant	28.0	6.2 (4)	Young + Old	Nubra	All	-0.25***	Elevation (−)***, Distance from glacier (+)***
*Exophiala*	Plant	50.5	6.2 (4)	Young + Old	Nubra	All	-0.21**	Elevation (−)*, TPI (+)*, Aspect (cos) (−)*
*Claroideoglomus*	Plant	11.1	6.2 (4)	Young + Old	Nubra	All	-0.171*	Elevation (−)***, Aspect (cos) (−)*, Distance from glacier (+)***
*Papiliotrema*	Bulk Soil	46.6	6.2 (4)	Young + Old	Nubra	All	-0.153*	Distance from glacier (+)***

Taxa are ranked by the number of networks in which they were identified as high-centrality nodes (65 total networks). Environmental associations represent significant FDR-corrected relationships between genus relative abundance and environmental variables; the direction of relationships is indicated by the sign of Spearman’s rho (* *P*_adj_ < .05, ** < .01, *** < .001).

### Environmental factors influencing source colonization and microbial diversity

Structural equation modelling supported a successional pathway linking glacier distance, source colonization, soil development, and microbial diversity. The full model found four out of the nine direct effects to be significant (*P* < .05), while the effect of soil development on bacterial richness was marginally significant (*P* = .055) (Supplementary Table S10, Supplementary Fig. S9). Thus, five pathways were retained in the trimmed SEM (Supplementary Table S11). In the final trimmed partial-PCA (conditioned by location) model, increasing distance from the glacier was positively associated with source colonization [β (standardized regression coefficient) = 0.61, *P* < .001] (Fig. 7), while topographic position showed a weaker negative association with source colonization (β = −0.26, *P* = .036). Increasing source colonization was strongly associated with soil development (β = 0.73, *P* < .001), indicating that increasing establishment of sources such as vascular plants, mosses, and lichens were coupled with soil development across the glacier forefields. Soil development was positively related to bacterial metacommunity richness (β = 0.51, *P* < .001), whereas fungal metacommunity richness was primarily associated with source colonization (β = 0.51, *P* < .001). Together, the model explained 26% of bacterial richness and 26% of fungal richness variation. Comparisons of the full and trimmed model based on global model fit parameters is presented in the Supplementary Material (Appendix 1).

**Figure 7:**
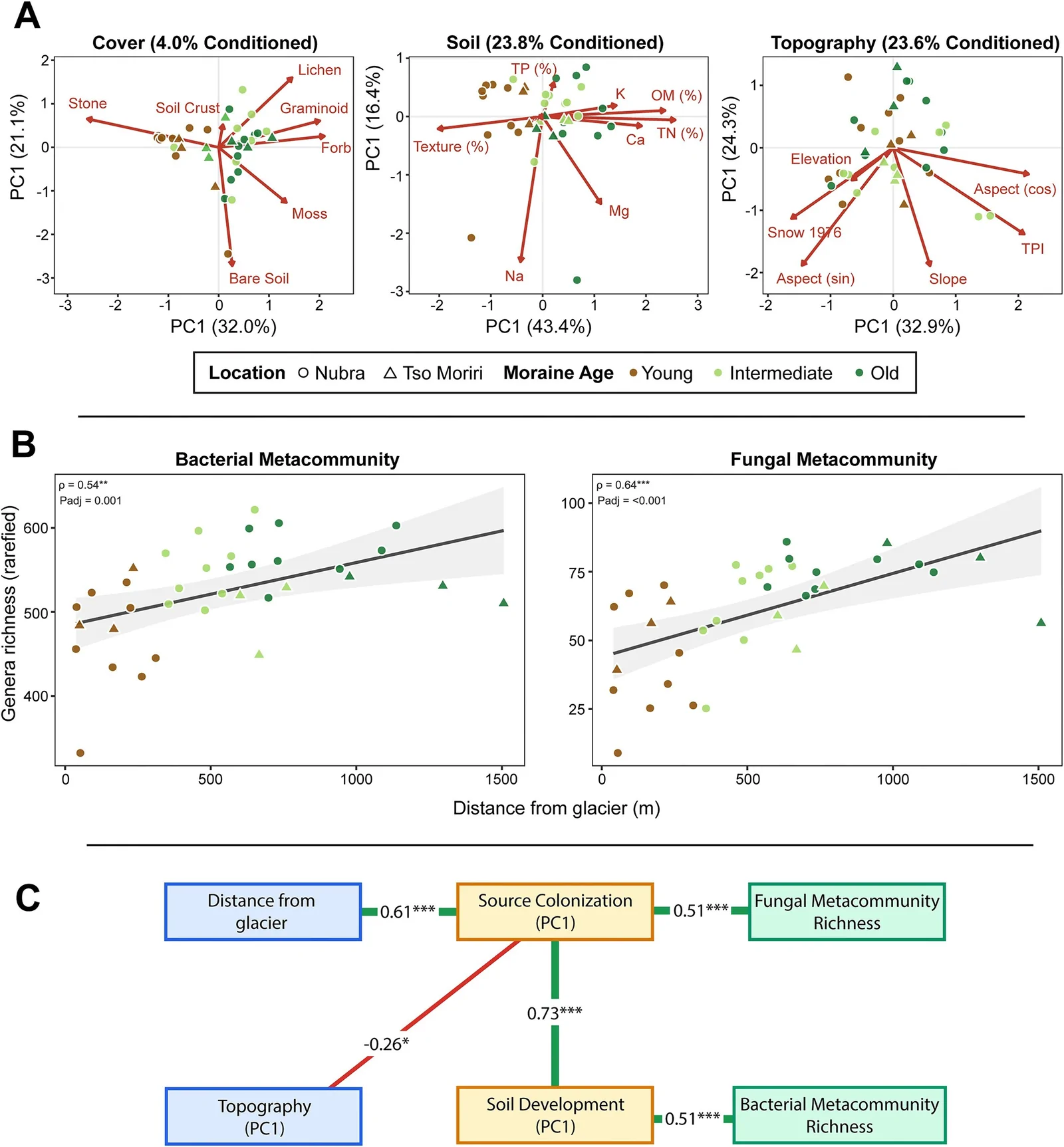
Integrated overview of the environmental drivers of microbial holobiome assembly. (A) Partial PCA ordinations of cover measurements, soil physicochemical properties, and topographic variables, conditioned by location across 36 plots. (B) Pearson correlations between distance from glacier and bacterial and fungal metacommunity genus richness, estimated from rarefied datasets across 999 iterations. (C) Final trimmed structural equation model illustrating relationships among primary abiotic factors, secondary abiotic factors, and biotic responses. All continuous variables were z-score transformed prior to modelling, and standardized path coefficients are shown.

The SEM results suggest distinct successional controls on bacterial and fungal diversity, with bacteria more strongly linked to soil development and fungi more directly associated with source establishment. Model sensitivity analyses showed that the core source colonization effects upon soil development pathway remained consistent after accounting for location effects, suggesting that successional changes across the glacier forefields are not solely driven by differences between glacier systems. When location was explicitly included, it explained additional variation in source colonization and soil development, indicating that the two glacier regions differ in baseline environmental conditions (Supplementary Fig. S10). Furthermore, tests including bacterial and fungal metacommunity Shannon H’ were non-significant and reported in the Supplementary Materials (Appendix 1, Supplementary Table S12).

## Discussion

As the initial colonizers and foundation of biogeochemical cycling in glacier forefields, understanding the influence of unique sources upon microbial holobiome assembly is paramount to preserving biodiversity and maintaining ecosystem stability over long-term succession. Bacterial and fungal communities are shaped by a multitude of interacting abiotic (e.g. time, topography, soil development) and biotic (host colonization) factors, while changing climate regimes is shifting colonization patterns within alpine regions (Wookey et al. 2009, Chen et al. 2025). This study extends the existing knowledge by addressing the importance of these hosts and abiotic sources (lichens, BSCs, mosses, bulk soil, and plants) in shaping microbial diversity and investigating their role in bacterial and fungal metacommunity assembly across wide environmental gradients of soil development, topography, and changing cover measurements at four glacier forefields in the high desert region of Ladakh, India (northwestern Himalaya). Using a multidisciplinary chronosequence approach, integrated with GIS-based terrain analysis, molecular sequencing, and classical transect sampling, we confirmed that moraine age is the overarching driver of source colonization, which subsequently influences soil development and holobiome diversity (Fig. 7). As hypothesized, microbial α- and β-diversity differed among sources, with lichens consistently supporting the lowest diversity, whereas plants, mosses, and bulk soil harboured increasingly diverse communities in older moraines. Our second hypothesis was only partially supported. Topographic variables had little direct influence on bacterial–fungal community congruence, but they were significantly associated with the abundance of several bacterial and fungal indicator taxa, suggesting that environmental heterogeneity shapes key community members even when overall cross-kingdom congruence remains stable. Our third hypothesis was strongly supported, as source exclusion consistently altered microbial co-occurrence network structure, whereas moraine age influenced relatively few network parameters. Finally, structural equation modelling revealed a successional pathway in which increasing glacier distance and topography influenced source colonization (primarily vegetation), which promoted soil development. Subsequently, while soil development was the primary driver of bacterial metacommunity richness, source colonization more strongly associated with fungal metacommunity richness.

### Source identity is the primary determinant of microbial community assembly

Source identity was the strongest determinant of bacterial and fungal diversity and community composition, supporting our first hypothesis that different hosts and substrates create distinct microbial niches. Although most genera were shared among sources, each source harboured characteristic communities that reflected differences in resource availability, environmental exposure, and host filtering (Fig. 2). Furthermore, bacterial–fungal congruence was significant in all sources except bulk soil, suggesting that these microbial communities are tightly coupled under harsh glacial conditions, while also highlighting the role of hosts in structuring microbial consortia.

Lichens and biological soil crusts (BSCs), often the earliest colonizers of glacier forefields, supported relatively low microbial diversity, reflecting strong environmental filtering under exposed, nutrient-poor conditions. Lichen microbiomes often showed the lowest number of unique genera and were dominated by stress-tolerant taxa such as *Acidiphilium, Sphingomonas*, and *Dioszegia*, consistent with previous studies from alpine and polar environments (Li et al. 2023b, Zhang et al. 2015, Tagirdzhanova et al. 2025). Increasing evidence suggests these microorganisms contribute to lichen fitness through nutrient provisioning and protection against pathogens and environmental stress, although many interactions remain poorly resolved (Cernava et al. 2017). In contrast, BSC communities showed greater successional changes despite similarly low diversity (Supplementary Table 2, 3), corroborating our previous observations of cyanobacterial succession in BSCs within these glacier forefields (Ruka et al. 2023). Dominant bacterial (*Sphingomonas, Blastocatella*) and fungal (Verrucariales) taxa were characteristic of arid BSCs (Bates et al. 2012, Foesel et al. 2013, Zhang et al. 2016, Borchhardt et al. 2019, Zhou et al. 2023, Liu et al. 2024), while increasing fungal diversity across moraine age suggests that BSC succession extends beyond cyanobacterial replacement. The prevalence of lichenized fungi (such as *Endocarpon* and *Polyblastia)*, which contribute to soil aggregation and desiccation resistance, further represents the importance of fungi in stabilizing BSCs during early ecosystem development (Zhang et al. 2017, Guang et al. 2025).

Mosses occupy diverse microhabitats within glacier forefields, from rock crevices to biological soil crusts and the bases of plants, while supporting distinct microbial communities despite lacking true roots (Bragina et al. 2012, Alvarenga and Rousk 2022; Ramakrishnan et al. 2025). Specifically, our results showed that moss-associated bacterial and fungal diversity shifted throughout successional stages (Fig. 2, Supplementary Table S3). Although dominant bacterial taxa resembled those in biological soil crusts, cyanobacteria were less abundant, suggesting that mosses replace cyanobacteria as the primary carbon source and may shape their microbiome through tissue chemistry (higher C: N) and secondary metabolites (Klarenberg et al. 2020; Valeeva et al. 2022). Moss fungal communities also shared taxa with soil crusts, particularly Verrucariales and Pleosporales, but were distinguished by the abundance of the moss-associated parasite *Lamprospora*, demonstrating that these host-specific interactions extend to extreme alpine conditions (Vega et al. 2019). Together, the high microbial diversity and strong bacterial–fungal congruence suggest that mosses act as important microbial reservoirs during succession, although their ecological role may be threatened by climate-driven declines in moisture availability (Wookey et al. 2009).

Bulk soil and rhizosphere communities represent contrasting components of glacier forefield microbial assembly. Bulk soil likely functions as a microbial reservoir within the holobiome (Chen et al. 2023), supporting high bacterial and fungal diversity despite extremely low biomass in young moraines, where failed sequencing attempts indicate strong early successional constraints (Fig. 2) (Jiang et al. 2018, Feng et al. 2023). Unlike other sources, bulk soil lacked significant bacterial–fungal Procrustes congruence after accounting for location, suggesting that cross-kingdom associations in this habitat are less constrained by host-mediated selection and more strongly influenced by abiotic heterogeneity. However, the presence of plant-associated bacteria, such as *Solirubrobacter* (Thermoleophilia), a potentially mobile and UV-resistant genus frequently associated with plant roots, suggests that bulk soil communities may also receive microbial inputs from surrounding vegetation (Ruka et al. 2023). Fungal communities in bulk soil contained a high proportion of taxa shared across sources, but also showed increased representation of Tremellales, particularly *Vishniacozyma*, a cold-adapted yeast isolated from glacial sediments in the Canadian High Arctic (Tsuji et al. 2019). Together, these patterns suggest that bulk soil communities represent a mixture of environmentally filtered taxa adapted to extreme conditions and taxa recruited from adjacent biological sources, reflecting the dual influence of abiotic constraints and surrounding host-associated habitats.

In contrast, rhizosphere communities represented strong host-driven selection, consistently contributing unique taxa across the chronosequence and commonly exhibiting higher fungal diversity than other sources such as lichens and BSCs (Fig. 2) (Kuzyakov and Blagodatskaya 2015). Rhizosphere diversity increased with moraine age, particularly among fungi, and communities were enriched in taxa such as Pleosporales and Helotiales, which include both saprotrophic decomposers and stress-tolerant symbionts (Kotilínek et al. 2017, Malicka et al. 2022, Hiiesalu et al. 2023, Hussain et al. 2024). Fungal endophytes and symbionts such as *Nothodactylaria* and *Exophiala*, alongside widespread genera like *Comoclathris* and *Alternaria*, highlight both host specificity and broad environmental tolerance (Maciá-Vicente et al. 2016, Li et al. 2024). Bacterial rhizosphere communities were characterized by increased Gammaproteobacteria, including *Steroidobacter*, a genus associated with plant hormone production and stress tolerance (Fahrbach et al. 2008, Valverde et al. 2016). Together, these patterns highlight a clear divergence between bulk soil as a relatively uncoupled, environmentally structured reservoir and rhizosphere communities as strongly host-filtered systems, emphasizing the central role of plants in structuring holobiome assembly across glacier forefields.

### Environmental heterogeneity modifies bacterial–fungal congruence rather than determining it directly

Topography in glacier forefields does not act independently of moraine age and soil development, making it difficult to disentangle their overlapping effects (Yamagata 2022, Masumoto et al. 2023). While previous studies have demonstrated topographic influences on individual taxonomic groups (Solon et al. 2021, Masumoto et al. 2023, Ruka et al. 2023), our analysis of bacterial–fungal interactions found limited direct effects. Instead, inter-kingdom congruence was significant across all sources except bulk soil, suggesting that host-associated habitats impose stronger constraints on bacterial–fungal associations than non-host environments. Structural equation modelling further indicated that topography primarily influences source colonization, which subsequently shapes microbial assembly and may mediate indirect microclimatic effects (Fig. 7). Even after accounting for differences between locations, a positive relationship between elevation and Procrustes residuals was observed, indicating reduced bacterial–fungal congruence at higher elevations, particularly in Tso Moriri. This pattern may reflect increasing environmental constraints at higher elevations, where reduced vegetation establishment and harsher microclimatic conditions limit the development of host-associated microbial niches. While higher congruence in plant-associated communities is consistent with strong coupling between plant-derived carbon inputs and bacterial–fungal interactions in the rhizosphere (Huang et al. 2024), Procrustes analyses are sensitive to sample size (*n* = 89), and the lower number of bulk soil samples (*n* = 21) may reduce statistical power. Furthermore, sensitivity analyses without location conditioning revealed significant bacterial–fungal congruence in bulk soil (Supplementary Table S6), highlighting the strong influence of spatially structured factors, including bedrock and soil chemistry, on these communities. Therefore, the absence of significant congruence in location-conditioned bulk soil analyses likely reflects the combined effects of reduced host filtering and stronger abiotic constraints. Together, these results reinforce the importance of host-mediated filtering while demonstrating that abiotic heterogeneity remains an important regulator of microbial interactions in non-host environments.

Although topography had little effect on whole-community bacterial–fungal congruence, relationships with indicator taxa suggest that environmental heterogeneity instead influences key microbial community members. Among bacterial indicator taxa, relative abundance generally declined on north-facing slopes, which also supported lower source colonization in the SEM and is consistent with previous observations of colder microclimates and reduced vegetation establishment under these conditions (Körner and Hiltbrunner 2026). In contrast, fungal indicator taxa exhibited stronger and more diverse environmental associations, with significant relationships to aspect, topographic position, slope, elevation, and distance from the glacier tongue. This greater environmental sensitivity suggests that key fungal taxa may play a larger ecological role during later successional stages, as increasing plant colonization creates more complex microbial habitats and moderates soil temperature and moisture extremes (Philippot et al. 2011, Donhauser and Frey 2018). Because fungal communities are known to be highly responsive to soil microclimate (Castaño et al. 2018), changes in the abundance of these key taxa may alter bacterial–fungal interactions without substantially changing overall community congruence. Thus, these findings indicate that topography influences glacier forefield holobiomes primarily by modifying source colonization and consequently affecting the abundance of ecologically important taxa rather than restructuring entire microbial communities.

### Source-specific interactions determine microbial network assembly across succession

Across network analyses, source exclusion had a more consistent effect than moraine age, revealing the importance of specific sources in structuring microbial interaction networks. Given the limited sample size available for individual networks, these analyses should be considered exploratory and interpreted primarily as comparative assessments of network structure rather than definitive representations of microbial interaction structure (Faust et al. 2015) (Supplementary Fig. S8). Nevertheless, the consistent responses observed across source exclusion treatments provide insights into how different microhabitats may contribute to microbial organization within the glacier holobiome. For bacterial metacommunities, source exclusion altered network structure in source-specific ways, indicating that distinct substrates contribute different ecological roles in maintaining microbial associations and connectivity across glacier forefields. Mosses appeared to promote connectivity among bacterial assemblages, whereas lichens and biological soil crusts disproportionately influenced network density and robustness (Fig. 5). Lower connectance following lichen and soil crust exclusion indicates that these early successional habitats contribute disproportionately to shared microbial associations, potentially acting as microbial inoculum or providing common taxa that connect communities across the forefield, consistent with the low proportion of unique taxa observed throughout the chronosequences. These patterns may reflect highly integrated microbial consortia within early successional habitats, particularly in soil crusts, where cyanobacteria contribute nitrogen fixation and carbon assimilation while heterotrophic bacteria support decomposition and nutrient mineralization (Belnap et al. 2003, Schmidt et al. 2025). In contrast, plants contributed more unique and potentially specialized interactions, shown by decreasing average path length when excluded from networks (Finck et al. 2026), consistent with stronger host filtering within rhizosphere communities. Together, these findings suggest that early successional substrates act as important reservoirs and connectors of microbial interactions, while later-colonizing hosts contribute more specialized network hubs.

Fungal network structure was also consistently affected by source exclusion, but fungal communities exhibited greater sensitivity to successional development than bacterial communities. Plant exclusion produced the strongest changes, highlighting the importance of rhizosphere habitats in organizing fungal interactions. The reduction in natural connectance following plant exclusion indicates that plant-associated habitats support a greater proportion of shared fungal associations (Shen et al. 2022), while increased node dissimilarity suggests that removal of plant-derived niches restructures fungal community organization. In addition, lower average path length and higher edge connectance in older moraines indicate that fungal networks become increasingly interconnected as vegetation establishment and soil development progress. Furthermore, these findings corroborate similar increases in fungal network connectivity during later successional stages, as observed in glacier forefields in Antarctica (Dong et al. 2022, Vimercati et al. 2022). Collectively, these results suggest that fungal co-occurrence networks are shaped by both source-specific interactions and long-term successional processes, with plant colonization acting as a major driver of fungal network assembly. More importantly, our multi-source approach expands upon previous studies that primarily examined bulk soils, revealing additional interactions associated with host and substrate communities that may represent a substantial component of glacier forefield holobiome structure.

### Source colonization links glacier forefield succession to microbial diversity

Overall, our results indicate that microbial holobiome assembly in Himalayan glacier forefields is governed primarily by source colonization, while environmental succession acts indirectly through source establishment and soil development (Bradley et al. 2014). Rather than directly restructuring microbial communities, glacier retreat promotes the expansion of source-associated habitats that increasingly organize bacterial and fungal metacommunities, strengthen cross-kingdom congruence, and reshape co-occurrence networks. Structural equation modelling further demonstrated that topographic and successional effects are mediated through colonization, with increasing plant, lichen, and moss cover promoting soil development, which in turn drives bacterial richness, while increasing host cover more directly supports fungal richness (Chen et al. 2017, Ruka et al. 2023). These findings identify source identity as the ecological bridge linking landscape succession with microbial community assembly.

The different source types also represent complementary ecological niches throughout succession. Early colonizers, including biological soil crusts and lichens, provide habitats that support microbial establishment and contribute to holobiome assembly in young moraines. Thus, soil crusts may represent important microbial interaction environments that facilitate bacterial community connectivity, whereas lichens support more distinct microbial assemblages associated with exposed rock surfaces (Schmidt et al. 2025). As succession progresses, mosses become increasingly prevalent across diverse microhabitats, supporting high-diversity microbial communities and maintaining connectivity among microbial assemblages (Ramakrishnan et al. 2025), whereas plant rhizospheres promote stronger host-associated filtering, support fungal indicator taxa, and contribute to increasingly specialized microbial associations. These contrasting source-specific strategies highlight the importance of often-overlooked microhabitats in organizing microbial metacommunities during glacier forefield succession, with important implications under ongoing climate change. Glacier retreat is expected to accelerate vascular plant expansion while simultaneously altering the distribution and persistence of cryptogam-dominated habitats through warming, changing moisture regimes, and increased competition (Wookey et al. 2009, Praeg et al. 2025). Our results suggest that such shifts in source availability may reorganize the microbial holobiome by modifying the relative contribution of early-successional habitats and increasing the influence of host-associated rhizosphere communities. Consequently, climate-driven changes in source composition may alter microbial diversity and the structure of cross-kingdom associations during primary succession.

### Limitations and future directions

This study employed a novel source-based sampling strategy designed to capture the natural heterogeneity of microbial habitats across glacier forefields. Consequently, source representation was inherently uneven, reflecting patterns of natural colonization rather than balanced experimental sampling. Although this reduced statistical power for analyses such as Procrustes tests and co-occurrence networks, our statistical framework incorporated sensitivity analyses and complementary approaches to assess the robustness of the observed patterns. In addition, moraine age was classified using observable geomorphological features and distance from the glacier rather than direct chronologies, limiting our ability to resolve fine-scale successional dynamics. However, by inferring from geochronological studies in the region, the moraine ages in the two study locations are likely similar, apart from the oldest moraines which are likely >1000 years old in Nubra while potentially being >7000 years old in Tso Moriri due to a more arid climate. Furthermore, the logistical constraints associated with the Tso Moriri region, being extremely remote and higher elevation, also restricted sampling to a single transect, resulting in location-conditioned analyses to account for this imbalance.

Our findings demonstrate the value of adopting a holobiome perspective that explicitly incorporates multiple biotic sources when investigating microbial succession. Future studies should extend this approach to additional glacier forefields, particularly in understudied mountain systems such as the Himalaya and Andes, where glacier retreat has major ecological and societal consequences. Integrating repeated seasonal sampling with higher-resolution genomic approaches, including metagenomics or metatranscriptomics, would further clarify the roles of microbial activity, dormancy, functional potential, and inter-kingdom interactions throughout succession. Such approaches will improve our understanding of how host-associated habitats and environmental heterogeneity jointly regulate microbial community assembly and holobiome dynamics in rapidly changing alpine ecosystems.

## Supplementary Material

fiag107_Supplemental_File
