# Supplementary material for: Holobiomes in succession: post-glacial microbial communities are structured by hosts, time, and habitat heterogeneity
Source: FEMS Microbiol Ecol. 2026 Sep 28;102(11):fiag107. doi: 10.1093/femsec/fiag107 (PMC13695152; doi:10.1093/femsec/fiag107)
Supplement: Supplementary file 1 [file fiag107_supplemental_file.docx]

# Supplementary Material

# Appendix 1

# Supplementary Methods

### Moss and lichen identification

Mosses were identified at the University of South Bohemia in České Budějovice, Czechia, using a combination of classical morphological methods and molecular barcoding. This approach was necessary due to the limited and often inadequate taxonomic literature available for many moss groups in the region. DNA isolation, PCR amplification, and sequencing followed the protocols described in Kučera et al. (2019). The NaOH method was used for total genomic DNA extraction (Werner et al., 2002). Crude extracts were 10x diluted (chloroplast loci amplification) or 100x (ITS amplification) with 100 mM Tris-HCl (pH 8.3). For polymerase chain reactions, 0.5 μl DNA solution (10 μl final volume) was mixed with Plain PP MasterMix kit (Top-Bio, Vestec, Czech Republic) for normally amplifying samples and TP 2× Master Mix (Top-Bio) for amplification of ITS templates. Molecular identification was subsequently based on reference sequences from GenBank, targeting the nuclear ITS region, the plastid *trnL–trnF* intron and spacer, and/or the *rps4* gene.

Lichen identification was performed at Charles University in Prague, Czechia. Total genomic DNA was extracted directly from the lichen thallus using the cetyltrimethylammonium bromide (CTAB) protocol (Cubero et al., 1999). The fungal internal transcribed spacer (ITS) region (ITS1-5.8S-ITS2 rDNA) was amplified using the primers ITS1F (5’-CTT GGT CAT TTA GAG GAA GTA A-3’) (Gardes and Bruns, 1993) and ITS4 (5’-TCC TCC GCT TAT TGA TAT GC-3’) (White et al., 1990). Polymerase chain reactions (PCRs) were performed in 10 μl reaction volumes consisting of 6.5 μl Milli-Q water, 2 μl MyTaq reaction buffer, 0.1 μl MyTaq DNA polymerase (Bioline), 0.2 μl of each primer (25 mM), and 1 μl of template DNA. Successful PCR products were purified using AMPure XP SPRI paramagnetic beads (Beckman Coulter), following the manufacturer’s protocol. Sequencing was performed using the same ITS1F and ITS4 primers. The identity of the mycobiont was verified through sequence similarity searches using the BLAST tool (NCBI).

### Procrustes analysis

Location-conditioned ordinations were generated from genus-level CLR-transformed abundance matrices using redundancy analysis with the model structure clr ~ 1 + Condition(Location), thereby removing between-location variation while retaining within-landscape community structure. Procrustes correlation (r) and permutation-based significance tests (999 permutations) were used to assess bacterial–fungal congruence across all samples and separately among sources (lichen, biological soil crust, moss, bulk soil, and plant). As a sensitivity analysis, the same workflow was repeated without conditioning by location (clr ~ 1) to evaluate the influence of geographic variation on observed bacterial–fungal relationships (Supplementary Table 6).

Sample-level Procrustes residual distances were extracted from each analysis, with higher values indicating greater bacterial–fungal decoupling. Residuals were rank-transformed and related to environmental variables (TPI, slope, aspect [sine and cosine], elevation, Snow 1976 extent, and distance from the glacier tongue) using Spearman rank correlations. Distance from the glacier tongue was used as a continuous proxy for successional position along the chronosequence. The potential influence of repeated sampling within plots was assessed by testing Plot ID effects using ANOVA.

Procrustes-associated indicator genera were identified by correlating genus-level relative abundance with sample-level Procrustes residuals separately for each marker (16S rRNA and ITS2) and source. Genera were retained when they showed significant associations (p < 0.05) and reached a maximum relative abundance >1% within the corresponding source. Relationships between retained genera and environmental predictors identified from the residual analyses were subsequently tested using Spearman rank correlations, with p-values corrected using false discovery rate (FDR) correction within each genus and source.

### Co-occurrence network analysis

Network analyses were performed on GlacierID–Moraine units, defined by the combination of glacier identity and moraine age class (young, intermediate, old). Because young moraines on the two adjacent Nubra glacier tongues (*Nubra_1_A* and *Nubra_2_A*) each contained fewer than eight samples, these units were merged (*Nubra_1_2_A*) prior to network construction. Networks were constructed in NetCoMi using Spearman correlations on CLR-transformed abundance data with zero replacement (pseudocount = 1) and sparsification using the default correlation threshold (0.3). For fungal networks, all genera per unit were retained, whereas bacterial networks retained the 150 most frequent genera (highestFreq = 150). Hierarchical clustering was used to calculate global network parameters, including average dissimilarity (avDiss), average path length (avPath), clustering coefficient (clustCoef), modularity, edge connectivity, natural connectivity, network density, and positive edge percentage (PEP), as well as node-level centrality measures (degree, betweenness, closeness, and eigenvector centrality). Average dissimilarity and path length describe overall network connectedness, with lower values indicating more integrated networks. Clustering coefficient reflects local connectivity among taxa, whereas modularity describes the degree of network compartmentalization into modules. Edge connectivity and natural connectivity quantify structural robustness by estimating resistance to edge or node removal and the redundancy of alternative network pathways, respectively. Network density represents the proportion of realized associations relative to all possible associations, while PEP indicates the proportion of positive associations within the network (https://netcomi.de/reference/netanalyze).

To account for unequal sample sizes, GlacierID–Moraine units containing more than eight samples were analysed using ten independent iterations, each based on a random subsample of eight samples without replacement. Units containing exactly eight samples were analysed once without subsampling, and global and node-level parameters were averaged across iterations. Bootstrap variability (standard deviation of global parameters across iterations) is presented in Supplementary Figure 8. Genera with an eigenvector centrality ≥ 0.95 were classified as high-centrality taxa within individual networks. To evaluate the contribution of individual habitats to network topology, six source treatments were analysed for each GlacierID–Moraine unit: all sources combined and five leave-one-source-out networks excluding lichens, mosses, biological soil crusts, bulk soil, or vascular plants.

As a sensitivity analysis, co-occurrence networks were additionally constructed using the same parameters described above but with a broader grouping strategy, where all plots within each moraine age class were combined to generate networks containing ≥20 samples. Variation in global and node-level network parameters between the original 8-sample networks and larger 20-sample networks was then assessed to evaluate the sensitivity of network patterns to sample size and network delineation (Supplementary Figure 8).

# Supplementary Results

## Taxonomic description of bacterial and fungal communities within sources

The composition of bacterial communities varied across sources, particularly at higher taxonomic levels (Supplementary Figure 6 & 7). Lichens were dominated by Alphaproteobacteria (*Acidiphilium*, *Sphingomonas*), Bacteroidiia (*Hymenobacter*, *Spirosoma*), and Actinobacteria (*Friedmanniella*). Soil crusts showed high abundances of Cyanobacteria (*Nostoc*, *Tychonema*, *Aliterella*), Blastocatellia (*Blastocatella*), and Alphaproteobacteria (*Sphingomonas*). Moss-associated communities were highly diverse, with dominant genera from Blastocatellia (*Blastocatella*, *RB41*), Thermoleophilia (*Solirubrobacter*), Cyanobacteria (*Nostoc*), and Alphaproteobacteria (*Sphingomonas*). Bulk soil communities were abundant in Thermoleophilia (*Solirubrobacter*, *Gaiella*) and Vicinamibacteria (unidentified genera), while Bacteroidiia were less abundant. Rhizosphere communities of plants had higher proportions of Gammaproteobacteria (*Steroidobacter*) and Polyangia (unidentified genera), along with persistent abundances of Alphaproteobacteria (*Sphingomonas*) and Actinobacteria (*Nocardioides*, *Actinoplanes*). Overall, these findings indicate broad taxonomic shifts among sources, yet certain genera, such as *Sphingomonas* and *Blastocatella*, remain consistently abundant across multiple sources.

Fungal communities exhibited strong specificity to sources, with substantial variation at the order level. In lichens, symbiotic fungi from the orders Teloschistales (*Xanthoria*) and Lecanorales (*Lecidella*, *Rhizoplaca*) were commonly abundant. Additionally, other fungal taxa often co-occurred with the primary symbionts, including *Knufia* (Chaetothyriales) and *Dioszegia* (Tremellales). Soil crust communities were primarily dominated by Verrucariales (*Endocarpon*, *Atla*, *Polyblastia*, *Verrucaria*), with notable contributions from Pleosporales (*Alternaria*) and Helotiales (*Tetracladium*). Moss-associated fungal communities were rich in Pleosporales (*Comoclathris*, *Phaeosphaeria*), Verrucariales (*Endocarpon*, *Agonimia*, *Polyblastia*, *Atla*), and Pezizales (*Lamprospora*). Bulk soil fungal communities also showed high abundances of Pleosporales (*Comoclathris*, *Alternaria*, *Cohesyomyces*, *Paraphoma*) and Verrucariales (*Endocarpon*), alongside elevated abundances of Tremellales (*Vishniacozyma*). In plant rhizosphere communities, dominant fungal taxa included Pleosporales (*Comoclathris*, *Alternaria*, *Darksidea*, *Paraphoma*), Helotiales (*Tetracladium*, *Cistella*), Chaetothyriales (*Exophiala*, *Cyphellophora*, *Fonsecaea*), and Xylariales (*Nothodactylaria*). As with bacterial communities, certain fungal taxa, such as *Endocarpon* and *Comoclathris*, were broadly distributed and consistently abundant across multiple sources, despite significant taxonomic shifts at the order level.

## Structural Equation Models

The full richness SEM included nine direct pathways. Global fit was mixed relative to conventional cutoffs: χ² was non-significant and CFI indicated good comparative fit, but RMSEA exceeded the ≤ 0.08 criterion (χ² = 5.61, df = 4, *p* = 0.231; CFI = 0.984; RMSEA = 0.106; SRMR = 0.057). The trimmed model retaining pathways with *p* ≤ 0.1 (five direct pathways) showed a similar pattern (χ² = 11.61, df = 8, *p* = 0.169; CFI = 0.964; RMSEA = 0.112; SRMR = 0.126), with slightly lower information criteria than the full model (AIC = 321.1 vs. 323.1; BIC = 336.9 vs. 345.3). Given the small model *df*, we interpret the richness SEM primarily through standardized path coefficients (Supplementary Tables 10–11) rather than as uniformly well-fitting by all absolute indices.The retained pathways represented the primary successional relationships: distance → source colonization, source colonization → soil development, soil development → bacterial richness, and source colonization → fungal richness.

Structural equation modelling of Shannon diversity (H′) used the same partial-PCA specification (location-conditioned composites; nine direct pathways; no direct environmental paths to diversity outcomes). Fit indices met the stated cutoffs (χ² = 3.11, df = 4, *p* = 0.540; CFI = 1.000; RMSEA = 0.000; SRMR = 0.043); however, with only four degrees of freedom RMSEA is zero whenever χ² < df and is not, on its own, strong evidence of substantive fit. Bacterial Shannon H′ increased with soil development (β = 0.45, p = 0.111), and fungal Shannon H′ was most strongly associated with source colonization (β = 0.34, p = 0.149), with a weaker soil pathway (β = 0.28, p = 0.290); none of the direct diversity paths reached conventional significance (p < 0.05). The model explained 23% of bacterial Shannon H′ variance and 33% of fungal Shannon H′ variance (R² = 0.23 and 0.33, respectively), compared with 43% and 55% for the vegetation and soil composites. Together, these results support the distance → colonization → soil successional sequence, but indicate that environmental and successional gradients were more strongly linked to the total number of taxa (richness) than to community evenness (Shannon H′).

# Appendix 2

## Supplementary Tables and Figures

### **Supplementary Table 1.** Identification and distribution of sequenced microbial community samples across moraine ages, locations, and sources.

| **Source** | **Young** | | | **Intermediate** | | | **Old** | | | **Grand Total** |
| --- | --- | --- | --- | --- | --- | --- | --- | --- | --- | --- |
|  | **NUB** | **TSO** | **Total** | **NUB** | **TSO** | **Total** | **NUB** | **TSO** | **Total** |  |
| **Bulk Soil** | 3 |  | **3** | 7 | 3 | **10** | 6 | 2 | **8** | **21** |
|  |  |  |  |  |  |  |  |  |  |  |
| **Soil Crust** | 9 | 3 | **12** | 8 | 3 | **11** | 9 | 3 | **12** | **35** |
|  |  |  |  |  |  |  |  |  |  |  |
| **Lichen** | 3 | 1 | **4** | **1**4 | **5** | **19** | 10 | 3 | **13** | **36** |
| *Xanthoria elegans* | 2 | 1 | **3** | 6 | 3 | **9** | 4 | 2 | **6** | 18 |
| *Gyalolechia bracteata/desertorum* |  |  |  | 4 |  | **4** |  |  |  | 4 |
| *Lecidea tessellata* |  |  |  |  |  |  | 2 |  | **2** | 2 |
| *Aspicilia* sp. |  |  |  |  |  |  | 1 |  | **1** | 1 |
| *Lecanora* sp. |  |  |  | 1 |  | **1** |  |  |  | 1 |
| *Lecidella stigmatea/patavina* |  |  |  |  |  |  | 1 |  | **1** | 1 |
| *Rhizoplaca melanophtalma* |  |  |  | 1 |  | **1** |  |  |  | 1 |
| Unidentified | 1 |  | **1** | 2 | 2 | **4** | 2 | 1 | **3** | 8 |
|  |  |  |  |  |  |  |  |  |  |  |
| **Moss** | 6 |  | **6** | 10 |  | **10** | 10 | 2 | **12** | **28** |
| *Distichium capillaceum* | 1 |  | **1** | 6 |  | **6** | 2 | 1 | **3** | 10 |
| *Stegonia latifolia* | 1 |  | **1** |  |  |  | 2 |  | **2** | 3 |
| *Tortella alpicola* | 1 |  | **1** | 1 |  | **1** |  |  |  | 2 |
| *Bryum argenteum* |  |  |  |  |  |  |  | 1 | **1** | 1 |
| *Bryum* spp. | 2 |  | **2** | 1 |  | **1** | 4 |  | **4** | 7 |
| *Didymodon* spp. |  |  |  |  |  |  | 1 |  | **1** | 1 |
| *Myurella julacea* |  |  |  | 1 |  | **1** |  |  |  | 1 |
| *Orthothecium strictum* |  |  |  | 1 |  | **1** |  |  |  | 1 |
| *Buckia vaucheri* |  |  |  |  |  |  | 1 |  | **1** | 1 |
| *Syntrichia* spp. | 1 |  | **1** |  |  |  |  |  |  | 1 |
|  |  |  |  |  |  |  |  |  |  |  |
| **Plant** | 15 | 8 | **23** | 27 | 8 | **35** | 26 | 10 | **36** | **94** |
| *Carex borii* |  |  |  | 8 |  | **8** | 3 |  | **3** | 11 |
| *Waldheimia tridactylites* | 3 | 2 | **5** | 1 |  | **1** |  | 3 | **3** | 9 |
| *Thylacospermum caespitosum* |  | 3 | **3** | 2 | 2 | **4** |  | 1 | **1** | 8 |
| *Saussurea glacialis* |  | 1 | **1** | 3 | 2 | **5** |  | 1 | **1** | 7 |
| *Carex sagaensis* |  |  |  | 2 |  | **2** | 4 |  | **4** | 6 |
| *Potentilla pamirica* |  |  |  |  | 1 | **1** | 5 |  | **5** | 6 |
| *Sibbaldia tetrandra* |  |  |  | 3 |  | **3** | 2 |  | **2** | 5 |
| *Poa attenuata* |  | 1 | **1** | 1 |  | 1 |  | 3 | **3** | 5 |
| *Carex nivalis* | 4 |  | **4** |  |  |  |  |  |  | 4 |
| *Kobresia schoenoides* |  |  |  | 1 |  | **1** | 3 |  | **3** | 4 |
| *Leontopodium ochroleucum* |  |  |  | 1 |  | **1** | 3 |  | **3** | 4 |
| *Waldheimia nivalis* | 3 |  | **3** |  |  |  |  |  |  | 3 |
| *Astragalus confertus* |  |  |  | 1 |  | **1** | 2 |  | **2** | 3 |
| *Arenaria bryoides* |  |  |  |  | 2 | **2** |  |  |  | 2 |
| *Bistorta vivipara* |  |  |  |  |  |  | 1 |  | **1** | 1 |
| *Dasiphora dryadanthoides* |  |  |  | 1 |  | **1** |  |  |  | 1 |
| *Draba altaica* | 1 |  | **1** |  |  |  |  |  |  | 1 |
| *Draba lasiophylla* | 1 |  | **1** |  |  |  |  |  |  | 1 |
| *Elymus jacquemontii* |  |  |  |  |  |  | 1 |  | **1** | 1 |
| *Kobresia pygmaea* |  |  |  |  | 1 | **1** |  |  |  | 1 |
| *Lloydia serotina* |  |  |  | 1 |  | **1** |  |  |  | 1 |
| *Nepeta longibracteata* | 1 |  | **1** |  |  |  |  |  |  | 1 |
| *Oxytropis platysema* |  |  |  |  |  |  | 1 |  | **1** | 1 |
| *Saussurea gnaphaloides* |  |  |  |  |  |  |  | 1 | **1** | 1 |
| *Saxifraga nanella* |  |  |  |  |  |  |  | 1 | **1** | 1 |
| *Saxifraga pulvinaria* |  |  |  | 1 |  | **1** |  |  |  | 1 |
| *Silene gonosperma* | 1 |  | **1** |  |  |  |  |  |  | 1 |
| *Stellaria decumbens* |  | 1 | **1** |  |  |  |  |  |  | 1 |
| *Tanacetum spp.* |  |  |  | 1 |  | **1** |  |  |  | 1 |
| *Thalictrum alpinum* |  |  |  |  |  |  | 1 |  | **1** | 1 |
| *Urtica hyperborea* | 1 |  | **1** |  |  |  |  |  |  | 1 |

### **Supplementary Table 2.** Analysis of variance (Type II F-test) results for α-diversity metrics of fungal and bacterial communities across categorical moraine ages and different sources. Models were fit using linear mixed-effects models with Plot ID included as a random effect. Significance was assessed using Type II F-tests to evaluate the contribution of fixed effects with corrected degrees of freedom.

| **Explanatory Variable** |  | **Bacteria** | | | | **Fungi** | | | |
| --- | --- | --- | --- | --- | --- | --- | --- | --- | --- |
|  |  | **Chao Richness** | | **H’ Index** | | **Chao Richness** | | **H’ Index** | |
|  | **Df** | **F** | **p** | **F** | **p** | **F** | **p** | **F** | **p** |
| Moraine | 2 | 6.35 | **0.005** | 4.77 | **0.016** | 10.09 | **<0.001** | 7.12 | **0.003** |
| Source | 4 | 47.34 | **<0.001** | 75.51 | **<0.001** | 66.29 | **<0.001** | 34.48 | **<0.001** |
| Location | 1 | 0.27 | 0.605 | 0.65 | 0.428 | 0.00 | 0.962 | 0.43 | 0.517 |
| Moraine:Source | 8 | 1.49 | 0.163 | 1.25 | 0.272 | 2.41 | 0.017 | 1.68 | 0.107 |
| Moraine:Location | 2 | 2.89 | 0.070 | 1.57 | 0.222 | 0.53 | 0.595 | 0.86 | 0.434 |
| Source:Location | 4 | 2.44 | **0.049** | 1.65 | 0.164 | 4.44 | **0.002** | 1.05 | 0.384 |
| Moraine:Source:  Location | 6 | 0.96 | 0.443 | 0.53 | 0.755 | 1.86 | 0.092 | 2.33 | **0.035** |

### **Supplementary Table 3.** PERMANOVA results for bacterial and fungal community composition across moraines of different categorical ages and locations (Nubra and Tso Moriri), analyzed for all samples combined and separately by source. Community data were CLR-transformed and Euclidean distances were used for the analysis. R² values indicate the proportion of variance explained. Significance is indicated by asterisks (*p_adj_ < 0.05, ** < 0.01). Plot was included as a blocking factor in the combined analysis, and as a fixed factor in separate analyses when applicable.

| **Source** | | **N** | **Total Variation** | **R^2^ (F)** | | | |
| --- | --- | --- | --- | --- | --- | --- | --- |
|  |  |  |  | **Moraine** | **Source** | **Location** | **Plot** |
| **Bacteria** | All | 211 | 6,687,441 | **2.1 (2.88)**** | **20.9 (14.1)**** | **1.4 (3.77)**** | Block |
|  | All (+ Plot) | 211 | 6,687,441 | **2.1 (3.10)**** | **20.9 (15.15)**** | **1.4 (4.06)**** | **16.5 (1.49)**** |
|  | Lichen | 35 | 750,659 | 6.0 (0.87) | - | 4.1 (1.19) | 51.5 (0.74) |
|  | Soil Crust | 35 | 914,253 | **9.7 (1.75)*** | - | 4.0 (1.40) | - |
|  | Moss | 28 | 222,353 | **10.3 (1.87)*** | - | 3.6 (1.31) | 77.8 (1.35) |
|  | Bulk Soil | 21 | 551,067 | 10.4 (1.14) | - | **12.1 (2.66)*** | - |
|  | Plant | 92 | 232,4951 | **6.7 (3.76)**** | - | **3.9 (4.33)**** | **37.5 (1.40)**** |
| **Fungi** | All | 207 | 1,475,523 | **2.8 (3.29)**** | **10.0 (5.92)**** | **3.2 (7.62)**** | Block |
|  | All (+ Plot) | 201 | 1,475,523 | **2.8 (3.67)**** | **10.0 (6.62)**** | **3.2 (8.52)**** | **21.0 (1.73)**** |
|  | Lichen | 35 | 132,217 | 6.2 (1.01) | - | **6.7 (2.22)*** | 53.7 (0.88) |
|  | Soil Crust | 31 | 141,148 | **10.7 (1.70)*** | - | 4.4 (1.40) | - |
|  | Moss | 27 | 222,353 | **10.4 (1.56)*** | - | **6.7 (2.01)*** | 72.8 (1.09) |
|  | Bulk Soil | 23 | 246,713 | 9.5 (1.14) | - | **11.4 (2.74)**** | - |
|  | Plant | 91 | 586,623 | **6.2 (3.80)**** | - | **7.7 (9.42)**** | **39.8 (1.63)**** |

### **Supplementary Table 4.** Analysis of variance (Type II F-test) results for beta-dispersion (distance to centroid) metric of fungal and bacterial communities across categorical moraine ages and different sources. Models were fit using linear mixed-effects models with Plot ID included as a random effect. Significance was assessed using Type II F-tests to evaluate the contribution of fixed effects with corrected degrees of freedom.

| **Explanatory Variable** | **Distance to centroid** | | | | | |
| --- | --- | --- | --- | --- | --- | --- |
|  | **Bacteria** | | | **Fungi** | | |
|  | **Df** | **F** | **p** | **Df** | **F** | **p** |
| Moraine | 2 | 0.448 | 0.643 | 2 | 11.841 | <0.001 |
| Source | 4 | 3.538 | 0.008 | 4 | 54.251 | <0.001 |
| Location | 1 | 0.215 | 0.646 | 1 | 5.245 | 0.03 |
| Moraine:Source | 8 | 2.575 | 0.011 | 8 | 2.663 | 0.009 |
| Moraine:Location | 2 | 3.627 | 0.036 | 2 | 0.746 | 0.482 |
| Source:Location | 4 | 0.513 | 0.726 | 4 | 1.606 | 0.176 |
| Moraine:Source:  Location | 6 | 1.013 | 0.412 | 6 | 1.581 | 0.156 |

### **Supplementary Table 5.** Bacterial–fungal community congruence assessed using Procrustes analysis across Ladakh glacier forefield samples, including all samples combined and analyses conducted separately by source. Procrustes analyses were performed using location-conditioned CLR-transformed RDA ordinations (*clr ~ 1 + Condition(Location))* with symmetric Protest tests based on 999 permutations. Reported *p*-values represent nominal Protest significance values. Linear mixed-effect model (LME) analyses assessed relationships between rank-transformed Procrustes residuals and rank-transformed environmental predictors. The influence of plot identity was evaluated using ANOVA, with F-statistics reported. Asterisks indicate nominal significance (* *p* < 0.05).

| **Analysis** | **Metric** | **All** | **Lichen** | **Soil Crust** | **Moss** | **Bulk Soil** | **Plant** |
| --- | --- | --- | --- | --- | --- | --- | --- |
| Procrustes | N | 201 | 34 | 31 | 26 | 21 | 89 |
|  | R | 0.73 | 0.44 | 0.44 | 0.46 | 0.23 | 0.57 |
|  | P-value (FDR) | <0.001 | <0.001 | 0.01 | 0.02 | 0.63 | <0.001 |
| Linear Models | Distance from Glacier | -0.04 | 0.08 | 0.01 | -0.06 | 0 | -0.04 |
|  | Elevation | 0.15* | -0.24 | 0.09 | -0.25 | 0.08 | 0.01 |
|  | TPI | 0 | 0.26 | -0.25 | -0.08 | 0.23 | 0.05 |
|  | Aspect (Cosine) | -0.1 | -0.09 | -0.02 | -0.01 | -0.06 | -0.11 |
|  | Aspect (Sine) | 0.1 | -0.27 | -0.13 | -0.08 | 0.05 | 0.03 |
|  | Slope | 0.01 | -0.1 | -0.19 | -0.03 | 0.08 | 0 |
|  | Snow 1976 | 0.15 | 0.07 | -0.38 | 0.24 | -0.35 | 0.18 |

### **Supplementary Table 6.** Bacterial–fungal community congruence assessed using Procrustes analysis across Ladakh glacier forefield samples, including all samples combined and analyses conducted separately by source. Procrustes analyses were performed using unconstrained CLR-transformed RDA ordinations (*clr* ~ 1) with symmetric Protest tests based on 999 permutations. Reported *p*-values represent nominal Protest significance values. Linear mixed-effect model (LME) analyses assessed relationships between rank-transformed Procrustes residuals and rank-transformed environmental predictors. The influence of plot identity was evaluated using ANOVA, with F-statistics reported. Asterisks indicate nominal significance (* *p* < 0.05).

| **Analysis** | **Metric** | **All** | **Lichen** | **Soil Crust** | **Moss** | **Bulk Soil** | **Plant** |
| --- | --- | --- | --- | --- | --- | --- | --- |
| Procrustes | N | 201 | 34 | 31 | 26 | 21 | 89 |
|  | R | 0.71 | 0.5 | 0.48 | 0.5 | 0.5 | 0.61 |
|  | P-value (FDR) | <0.001 | <0.001 | <0.001 | <0.001 | 0.01 | <0.001 |
| Linear Models | Distance from Glacier | 0 | 0.27 | 0.03 | -0.06 | 0.05 | -0.10 |
|  | Elevation | 0.26* | -0.03 | 0.18 | 0.05 | 0.01 | 0.29* |
|  | TPI | -0.02 | 0.16 | -0.20 | -0.19 | 0.21 | -0.08 |
|  | Aspect (Cosine) | 0.02 | -0.01 | 0.03 | -0.10 | -0.16 | 0.16 |
|  | Aspect (Sine) | 0.14* | -0.11 | -0.02 | 0.19 | -0.03 | 0.13 |
|  | Slope | 0.05 | 0.02 | -0.15 | -0.08 | -0.11 | -0.03 |
|  | Snow 1976 | 0.05 | 0.12 | -0.35 | 0.32 | 0.10 | 0.12 |
| ANOVA (F statistic) | Plot | 0.79 | 0.84 | 0.81 | NA | NA | 1.16 |

### **Supplementary Table 7.** ANOVA results for global network parameters derived from bacterial and fungal community networks under separate source exclusion treatments. Data were analysed using linear mixed-effects models with GlacierID × Moraine unit included as a random effect. *P*-values were adjusted using false discovery rate (FDR) correction. Bold values indicate parameters selected for visualization in the main text based on χ² ranking (top three parameters). Only FDR-corrected *p*-values < 0.01 are visualized in the corresponding boxplots (Figures 5 and 6).

| **Network Parameter** | **Term** | **Bacteria** | | | **Fungi** | | |
| --- | --- | --- | --- | --- | --- | --- | --- |
|  |  | **X^2^** | **df** | ***p*_adj_** | **X^2^** | **df** | ***p*_adj_** |
| Avg. Node Dissimilarity | Exclusion treatment | 34.3 | 5 | <0.001 | **139.1** | **5** | **<0.001** |
|  | Moraine age | 7.89 | 2 | 0.077 | 3.07 | 2 | 0.287 |
|  | Treatment:Moraine | 17.7635 | 10 | 0.147 | 10.8398 | 10 | 0.376 |
| Avg. Path Length | Exclusion treatment | **40.9** | **5** | **<0.001** | 56.6 | 5 | <0.001 |
|  | Moraine age | 1.40 | 2 | 0.497 | **12.8** | **2** | **0.007** |
|  | Treatment:Moraine | 11.9412 | 10 | 0.348 | 17.5273 | 10 | 0.127 |
| Clustering Coefficient | Exclusion treatment | 36.1 | 5 | <0.001 | 101.4 | 5 | <0.001 |
|  | Moraine age | 5.14 | 2 | 0.189 | 0.48 | 2 | 0.785 |
|  | Treatment:Moraine | 19.3194 | 10 | 0.146 | 17.2606 | 10 | 0.127 |
| Modularity | Exclusion treatment | 24.1 | 5 | <0.001 | 27.3 | 5 | <0.001 |
|  | Moraine age | 2.59 | 2 | 0.313 | 5.01 | 2 | 0.164 |
|  | Treatment:Moraine | 13.1077 | 10 | 0.348 | 13.5982 | 10 | 0.256 |
| Edge Connectance | Exclusion treatment | 17.4 | 5 | 0.004 | 5.53 | 5 | 0.355 |
|  | Moraine age | 3.63 | 2 | 0.233 | **20.2** | **2** | **<0.001** |
|  | Treatment:Moraine | 11.7182 | 10 | 0.348 | 20.1865 | 10 | 0.110 |
| Natural Connectance | Exclusion treatment | **40.9** | **5** | **<0.001** | **182.3** | **5** | **<0.001** |
|  | Moraine age | 9.72 | 2 | 0.062 | 6.05 | 2 | 0.130 |
|  | Treatment:Moraine | 21.2330 | 10 | 0.146 | 10.7658 | 10 | 0.376 |
| Network Density | Exclusion treatment | 32.1 | 5 | <0.001 | 104.0 | 5 | <0.001 |
|  | Moraine age | 4.71 | 2 | 0.189 | 1.59 | 2 | 0.517 |
|  | Treatment:Moraine | 17.0500 | 10 | 0.147 | 16.7675 | 10 | 0.127 |
| Positive Edge Percentage | Exclusion treatment | **42.1** | **5** | **<0.001** | **114.5** | **5** | **<0.001** |
|  | Moraine age | 3.49 | 2 | 0.233 | 3.68 | 2 | 0.254 |
|  | Treatment:Moraine | 6.5914 | 10 | 0.763 | 24.4373 | 10 | 0.052 |

### **Supplementary Table 8.** Stability of bacterial indicator taxa identified by high node eigenvector centrality (>0.95) across networks constructed using sample sizes of 8 and 20. Indicator taxa were compared based on their frequency of detection as high-centrality nodes across network iterations.

| **Taxon** | **8 Sample Networks** | | | **20 Sample Networks** | | |
| --- | --- | --- | --- | --- | --- | --- |
|  | **No. of networks** | **%** | **Mean Eigenvalue Centrality** | **No. of networks** | **%** | **Mean Eigenvalue Centrality** |
| *Vicinamibacter* | 8/65 | 12.3 | 0.96 | 18/18 | 100 | 0.97 |
| Unclassified9_Subgroup 17(O) | 7/65 | 10.8 | 0.96 | 15/18 | 83.3 | 0.97 |
| Unclassified75_Pedosphaeraceae(F) | 5/65 | 7.7 | 0.97 | 10/18 | 55.6 | 0.97 |
| Unclassified8_Microscillaceae(F) | 4/65 | 6.2 | 0.96 | 9/18 | 50 | 0.97 |
| Unclassified1_67-14(F) | 4/65 | 6.2 | 0.97 | 6/18 | 33.3 | 0.97 |
| Unclassified89_OM190(C) | 4/65 | 6.2 | 0.96 | 4/18 | 22.2 | 0.95 |

### **Supplementary Table 9.** Stability of fungal indicator taxa identified by high node eigenvector centrality (>0.95) across networks constructed using sample sizes of 8 and 20. Indicator taxa were compared based on their frequency of detection as high-centrality nodes across network iterations.

| **Taxon** | **8 Sample Networks** | | | **20 Sample Networks** | | |
| --- | --- | --- | --- | --- | --- | --- |
|  | **No. of networks** | **%** | **Mean Eigenvalue Centrality** | **No. of networks** | **%** | **Mean Eigenvalue Centrality** |
| *Keissleriella* | 8/65 | 12.3 | 0.97 | 2/18 | 11.1 | 0.95 |
| Unclassified3_Glomeraceae(F) | 7/65 | 10.8 | 0.97 | 1/18 | 5.6 | 0.96 |
| Unclassified26_Diversisporaceae(F) | 5/65 | 7.7 | 0.97 | 2/18 | 11.1 | 0.96 |
| *Paraphoma* | 5/65 | 7.7 | 0.97 | - | - | - |
| *Claroideoglomus* | 4/65 | 6.2 | 0.99 | 1/18 | 5.6 | 0.96 |
| *Cyphellophora* | 4/65 | 6.2 | 0.99 | 1/18 | 5.6 | 0.96 |
| *Exophiala* | 4/65 | 6.2 | 0.99 | - | - | - |
| *Papiliotrema* | 4/65 | 6.2 | 0.99 | - | - | - |

### **Supplementary Table 10**. Standardized path coefficients for the full structural equation model (SEM) describing relationships among glacier succession, source colonization, soil development, and microbial richness. Continuous predictor variables and richness responses were standardized (z-score transformed) prior to analysis. *Estimate (raw)* represents the unstandardized regression coefficient, whereas *Beta (std)* represents the standardized path coefficient, allowing direct comparison of effect sizes among pathways. Standard errors (SE), 95% confidence intervals (CI), and associated *p*-values are shown. Rows in bold were retained in the trimmed model based on a < 0.1 p-value threshold.

| **Response** | **Predictor** | **β (std)** | **SE** | **95% CI** | ***p*** |
| --- | --- | --- | --- | --- | --- |
| **Source Colonization PC1** | **Distance from glacier** | **0.607** | **0.147** | **0.365, 0.951** | **<0.001** |
| **Source Colonization PC1** | **Topo PC1** | **-0.262** | **0.124** | **-0.491, 0.015** | **0.0356** |
| Soil development PC1 | Distance from glacier | 0.193 | 0.167 | -0.099, 0.548 | 0.2474 |
| Soil development PC1 | Topo PC1 | -0.065 | 0.150 | -0.436, 0.184 | 0.6626 |
| **Soil development PC1** | **Source colonization PC1** | **0.595** | **0.156** | **0.277, 0.908** | **0.0001** |
| Bacterial richness | Source colonization PC1 | 0.218 | 0.211 | -0.220, 0.588 | 0.3026 |
| **Bacterial richness** | **Soil development PC1** | **0.445** | **0.233** | **0.041, 0.939** | **0.0555** |
| **Fungal richness** | **Source colonization PC1** | **0.489** | **0.213** | **-0.050, 0.827** | **0.0216** |
| Fungal richness | Soil development PC1 | 0.170 | 0.228 | -0.240, 0.639 | 0.4557 |

### **Supplementary Table 11.** Standardized path coefficients for the final trimmed structural equation model (SEM) describing relationships among glacier succession, source colonization, soil development, and microbial richness. Continuous predictor variables and richness responses were standardized (z-score transformed) prior to analysis. *Estimate (raw)* represents the unstandardized regression coefficient, whereas *Beta (std)* represents the standardized path coefficient, allowing direct comparison of effect sizes among pathways. Standard errors (SE), 95% confidence intervals (CI), and associated *p*-values are shown.

| **Response** | **Predictor** | **β (std)** | **SE** | **95% CI** | ***p*** |
| --- | --- | --- | --- | --- | --- |
| Bacterial richness | Soil development (PC1) | 0.509 | 0.109 | 0.248, 0.693 | <0.001 |
| Fungal richness | Source colonization (PC1) | 0.511 | 0.130 | 0.196, 0.714 | <0.001 |
| Soil Development (PC1) | Source colonization (PC1) | 0.728 | 0.111 | 0.511, 0.941 | <0.001 |
| Source colonization (PC1) | Distance from glacier | 0.607 | 0.147 | 0.365, 0.951 | <0.001 |
| Source colonization (PC1) | Topo PC1 | -0.262 | 0.124 | -0.491, 0.015 | 0.036 |

### **Supplementary Table 12.** Standardized path coefficients for the full structural equation model (SEM) describing relationships among glacier succession, source colonization, soil development, and microbial Shannon diversity (H′). Continuous predictor variables and Shannon responses were standardized (z-score transformed) prior to analysis. Beta (std) represents the standardized path coefficient, allowing direct comparison of effect sizes among pathways. Standard errors (SE), 95% confidence intervals (CI), and associated *p*-values are shown (bootstrap BCa intervals, 1,000 resamples). Shannon H′ values are plot-level means across 999 rarefaction draws.

| **Response** | **Predictor** | **β (std)** | **SE** | **95% CI** | ***p*** |
| --- | --- | --- | --- | --- | --- |
| Bacterial Shannon H' | Source colonization PC1 | 0.037 | 0.252 | -0.563, 0.459 | 0.8837 |
| Bacterial Shannon H' | Soil development PC1 | 0.448 | 0.281 | -0.026, 1.055 | 0.1106 |
| Fungal Shannon H' | Source colonization PC1 | 0.337 | 0.234 | -0.282, 0.676 | 0.1489 |
| Fungal Shannon H' | Soil development PC1 | 0.28 | 0.264 | -0.114, 0.895 | 0.2897 |

### **Supplementary Table 13.** Plot-level surface cover (%) recorded in 1 m² quadrats across 36 plots in four glacier forefields (Nubra: NUB1–NUB3; Tsomoriri: TS). Cover categories include forbs, graminoids, lichens, mosses, biological soil crust, stones, and bare soil. Cover categories were estimated visually in the field.

|  |  |  |  |  |  | **Cover (%)** | | | | | | | |
| --- | --- | --- | --- | --- | --- | --- | --- | --- | --- | --- | --- | --- | --- |
| **Plot ID** | **Moraine age** | **Lat.** | **Long.** | **Elevation (m)** | **Distance** | **Forb** | **Graminoid** | **Lichen** | **Moss** | **Soil crust** | **Stones** | **Bare soil** |  |
| NUB1-A-1 | Young | 34.67503 | 77.75311 | 5404 | 42 | 0 | 0 | 0 | 0 | 40 | 45 | 15 |  |
| NUB1-A-2 | Young | 34.67457 | 77.75312 | 5408 | 94 | 5 | 0 | 0 | 0 | 10 | 85 | 0 |  |
| NUB1-A-3 | Young | 34.6734 | 77.75282 | 5399 | 227 | 4 | 1.5 | 0 | 0.5 | 4 | 90 | 0 |  |
| NUB1-B-1 | Intermediate | 34.67113 | 77.75213 | 5352 | 488 | 33.5 | 45 | 2 | 4 | 20 | 8 | 0 |  |
| NUB1-B-2 | Intermediate | 34.67036 | 77.75189 | 5353 | 573 | 72.5 | 6 | 9 | 0.5 | 33 | 8 | 0 |  |
| NUB1-B-3 | Intermediate | 34.6698 | 77.75117 | 5339 | 654 | 21.2 | 80 | 7 | 6 | 1.5 | 15 | 0 |  |
| NUB1-C-1 | Old | 34.66743 | 77.74966 | 5256 | 946 | 17.7 | 33 | 11.1 | 0.5 | 22 | 25 | 0 |  |
| NUB1-C-2 | Old | 34.6612 | 77.74949 | 5251 | 1090 | 23.2 | 17 | 1.6 | 2.5 | 10 | 8 | 37.7 |  |
| NUB1-C-3 | Old | 34.66572 | 77.74932 | 5244 | 1140 | 17.6 | 46 | 0 | 0.1 | 7 | 0 | 29.3 |  |
| NUB2-A-1 | Young | 34.66164 | 77.76261 | 5322 | 55 | 0 | 0 | 0 | 15 | 23 | 15 | 47 |  |
| NUB2-A-2 | Young | 34.66185 | 77.76141 | 5336 | 166 | 3.5 | 0 | 0 | 1 | 10 | 85 | 0.5 |  |
| NUB2-A-3 | Young | 34.66177 | 77.76028 | 5328 | 266 | 8 | 0 | 0 | 0.5 | 7.5 | 84 | 0 |  |
| NUB2-B-1 | Intermediate | 34.66161 | 77.7594 | 5303 | 358 | 10 | 0 | 0.7 | 4 | 5.5 | 80 | 0 |  |
| NUB2-B-2 | Intermediate | 34.66174 | 77.75888 | 5291 | 394 | 37.9 | 17.5 | 0.1 | 2 | 3 | 3 | 36.5 |  |
| NUB2-B-3 | Intermediate | 34.66187 | 77.75816 | 5280 | 461 | 10 | 36.6 | 6.5 | 3.5 | 15 | 8 | 20.4 |  |
| NUB2-C-1 | Old | 34.66199 | 77.75697 | 5261 | 569 | 31.6 | 35 | 4.5 | 0.5 | 4 | 6 | 18.4 |  |
| NUB2-C-2 | Old | 34.66133 | 77.75624 | 5247 | 635 | 43.5 | 25 | 4 | 3 | 3 | 16 | 5.5 |  |
| NUB2-C-3 | Old | 34.66111 | 77.75514 | 5236 | 737 | 56.6 | 60 | 0.1 | 1.5 | 2 | 15 | 0 |  |
| NUB3-A-1 | Young | 34.6529 | 77.74506 | 5141 | 40 | 2.5 | 17 | 0 | 0.7 | 80 | 0 | 0 |  |
| NUB3-A-2 | Young | 34.65303 | 77.74316 | 5147 | 214 | 14.1 | 11 | 4.5 | 0.7 | 10 | 60 | 0 |  |
| NUB3-A-3 | Young | 34.65289 | 77.74207 | 5139 | 314 | 5.5 | 10 | 1 | 0 | 10 | 70 | 3.5 |  |
| NUB3-B-1 | Intermediate | 34.65343 | 77.74178 | 5136 | 348 | 25.6 | 27 | 9 | 5 | 19 | 27 | 0 |  |
| NUB3-B-2 | Intermediate | 34.65413 | 77.74044 | 5106 | 483 | 11 | 21 | 23.5 | 3.5 | 27 | 34 | 0 |  |
| NUB3-B-3 | Intermediate | 34.6548 | 77.74008 | 5081 | 542 | 49.5 | 25 | 3.5 | 5 | 27 | 5 | 0 |  |
| NUB3-C-1 | Old | 34.6557 | 77.73941 | 5031 | 644 | 49.5 | 3 | 3 | 4 | 17 | 5 | 18.5 |  |
| NUB3-C-2 | Old | 34.65593 | 77.73888 | 5022 | 701 | 31.6 | 37 | 4.5 | 5 | 13 | 14 | 0 |  |
| NUB3-C-3 | Old | 34.65605 | 77.73853 | 5020 | 733 | 42 | 40 | 7 | 5 | 11 | 10 | 0 |  |
| TS-A-1 | Young | 32.98223 | 78.43537 | 5801 | 52 | 7.6 | 0.2 | 0 | 0 | 5 | 90 | 0 |  |
| TS-A-2 | Young | 32.98274 | 78.43423 | 5792 | 170 | 3.1 | 0 | 0 | 0 | 11 | 80 | 5.9 |  |
| TS-A-3 | Young | 32.9832 | 78.4337 | 5783 | 237 | 21.1 | 2.5 | 1 | 0 | 4 | 40 | 31.4 |  |
| TS-B-1 | Intermediate | 32.98527 | 78.43067 | 5802 | 604 | 9 | 0.5 | 7 | 0 | 8 | 60 | 15.5 |  |
| TS-B-2 | Intermediate | 32.9856 | 78.43005 | 5805 | 669 | 12 | 0 | 4.5 | 0 | 15 | 50 | 18.5 |  |
| TS-B-3 | Intermediate | 32.98637 | 78.42953 | 5794 | 763 | 2.8 | 30.1 | 8 | 0 | 20 | 45 | 0 |  |
| TS-C-1 | Old | 32.9868 | 78.42691 | 5821 | 980 | 36.3 | 30 | 8 | 5 | 10 | 10 | 0.7 |  |
| TS-C-2 | Old | 32.9883 | 78.42398 | 5839 | 1300 | 46.1 | 5 | 5 | 2.5 | 10 | 25 | 6.4 |  |
| TS-C-3 | Old | 32.9894 | 78.42214 | 5785 | 1509 | 44.6 | 3 | 3 | 1 | 6 | 40 | 2.4 |  |

### **Supplementary Figure 1.** Redundancy analysis (RDA) of soil physicochemical measurements as response variables and environmental parameters as explanatory variables across four glacier forefields in Ladakh, India. Soil variables were RMS-scaled prior to analysis. The model was conditioned by “Location” to account for spatial structure among sites.

### **Supplementary Figure 2.** Redundancy analysis (RDA) of plot cover measurements—including plants, moss, lichen, soil crust, bare soil, and stones—as response variables, with environmental parameters as explanatory variables, across four glacier forefields in Ladakh, India. Cover data were transformed using the isometric log-ratio (ILR) transformation. The model was conditioned by “Location” to account for spatial structure among sites.

### **Supplementary Figure 3.** Heatmap showing Spearman rank correlations between plot-level cover (%) measurements and topographic and environmental variables. Significance levels are based on FDR-corrected *p*-values: * *p*_adj_ < 0.05, ** < 0.01, *** < 0.001.

### **Supplementary Figure 4.** Heatmap showing Spearman rank correlations between topographic and environmental variables and soil physicochemical properties. Significance levels are based on FDR-corrected *p*-values: * *p*_adj_ < 0.05, ** < 0.01, *** < 0.001.

### **Supplementary Figure 5.** Heatmap showing Spearman rank correlations between plot-level cover (%) measurements and soil physicochemical properties. Significance levels are based on FDR-corrected *p*-values: * *p*_adj_ < 0.05, ** < 0.01, *** < 0.001.

### **Supplementary Figure 6.** Relative abundance of bacterial classes across moraine ages within each source. Samples were pooled by moraine age for each source. Colors represent the 15 most abundant bacterial classes, with all remaining classes grouped as Other.

### **Supplementary Figure 7.** Relative abundance of fungal orders across moraine ages within each source. Samples were pooled by moraine age for each source. Colors represent the 15 most abundant fungal orders, with all remaining orders grouped as Other.

### **Supplementary Figure 8.** Line graphs comparing global network parameters between 8-sample and 20-sample networks across moraine ages. Error bars represent ±1 SD of bootstrap subsampling variability. For the 8-sample Glacier × Moraine networks, standard deviations were calculated across 10 random 8-sample subsampling iterations for each network and then averaged across networks within each moraine age. For the 20-sample moraine-age networks, standard deviations were calculated across 10 random 20-sample subsampling iterations from the pooled samples within each moraine age. Moraine ages are denoted as young (A), intermediate (B), and old (C).

### **Supplementary Figure 9.** Full structural equation model (SEM) using partial PCA scores as predictor variables. Green arrows indicate positive relationships, red arrows indicate negative relationships, and faded arrows represent non-significant paths. Values along arrows are standardized path coefficients (β). Significance levels are indicated as * *p* < 0.05, ** < 0.01, and *** < 0.001.

### **Supplementary Figure 10.** Sensitivity analysis of the structural equation model (SEM) including location as an additional predictor. Green arrows indicate significant positive relationships, red arrows indicate significant negative relationships, and faded arrows represent non-significant paths. Values along arrows represent standardized path coefficients (β). Significance is indicated as * *p* < 0.05.
